# A review of the 661W cell line as a tool to facilitate treatment development for retinal diseases

**DOI:** 10.1186/s13578-025-01381-2

**Published:** 2025-04-01

**Authors:** Alicia A. Brunet, Rebekah E. James, Petria Swanson, Livia S. Carvalho

**Affiliations:** 1 Centre for Ophthalmology and Visual Science, The University of Western Australia, Nedlands, WA 6009 Australia; 2 Lions Eye Institute, 2 Verdun St, Nedlands, WA 6009 Australia; 3 School of Biomedical Sciences, The University of Western Australia, Crawley, WA 6009 Australia; 4 Department of Optometry and Vision Sciences, University of Melbourne, Parkville, VIC 3052 Australia

**Keywords:** 661W cells, Photoreceptor, Cell line, Retinal disease, Disease modelling

## Abstract

Retinal diseases encompass a diverse group of disorders that affect the structure and function of the retina, leading to visual impairment and, in some cases, irreversible vision loss. The investigation of retinal diseases is crucial for understanding their underlying mechanisms, identifying potential therapeutic targets, and developing effective treatments. The use of in vitro cell models has become instrumental in advancing our knowledge of these disorders, but given that these conditions usually affect retinal neuronal cell types, access to appropriate cell models can be potentially challenging. Among the available in vitro cell models, the 661W cone-like cell line has emerged as a valuable tool for studying various retinal diseases, ranging from monogenic conditions, such as inherited retinal diseases, to complex conditions such as age-related macular degeneration (AMD), diabetic retinopathy, amongst others. Developed from immortalized murine photoreceptor cells, and freely available for academics from its creator, the 661W cell line has offered visual scientists and clinicians around the world a reliable and well-characterised platform for investigating disease pathogenesis, exploring disease-specific molecular signatures, and evaluating potential therapeutic interventions. This review aims to provide an overview of the 661W cell line and its applications in the study of both inherited and acquired retinal diseases. By examining the applications and limitations of this unique cell line, we may gain valuable insights into its contributions in unravelling the complexities of retinal diseases and its potential impact on the development of novel treatments for these diseases.

Retinal diseases are a leading cause of vision impairment and blindness worldwide, with both inherited and acquired forms contributing to progressive degeneration of photoreceptors [1, 2]. Despite extensive research aimed at understanding the pathophysiological mechanisms underlying photoreceptor degeneration in various retinal diseases, exact mechanisms of photoreceptor loss require further exploration and effective treatments remain largely unavailable. The only currently available therapy Luxturna^®^, which is approved for a small subset of patients with inherited mutations in the *RPE65* gene [3], highlighting the need for further therapeutic developments.

Originally derived from retinal tumours in 1992, 661W cells are an immortalized cone photoreceptor-like line that have been characterized for their expression of cone photoreceptor markers and their ability to replicate certain aspects of retinal diseases, such as oxidative and metabolic stress [4, 5]. They have been used as models in the study of inherited and acquired retinal disease. Furthermore, their rapid proliferation and stable phenotype have facilitated their application in drug screening and gene therapy development. Though the use of 661W cells presents limitations, as the monolayer culture conditions are unable to replicate the complexity of the retinal architecture and microenvironment, amongst other physiological limiting factors. These limitations necessitate complementary approaches, whereby 661W cells are able to provide a platform for preliminary testing before further validation in more complex models, such as animal models, prior to clinical applications being considered. This review provides and in-depth examination of the characterization and diverse applications of the 661W cell line, highlighting its contributions to retinal disease research while addressing its limitations and future directions for its use in therapeutic development. An overview of the discussed topics within this review are provided in Fig. 1.

**Fig. 1:**
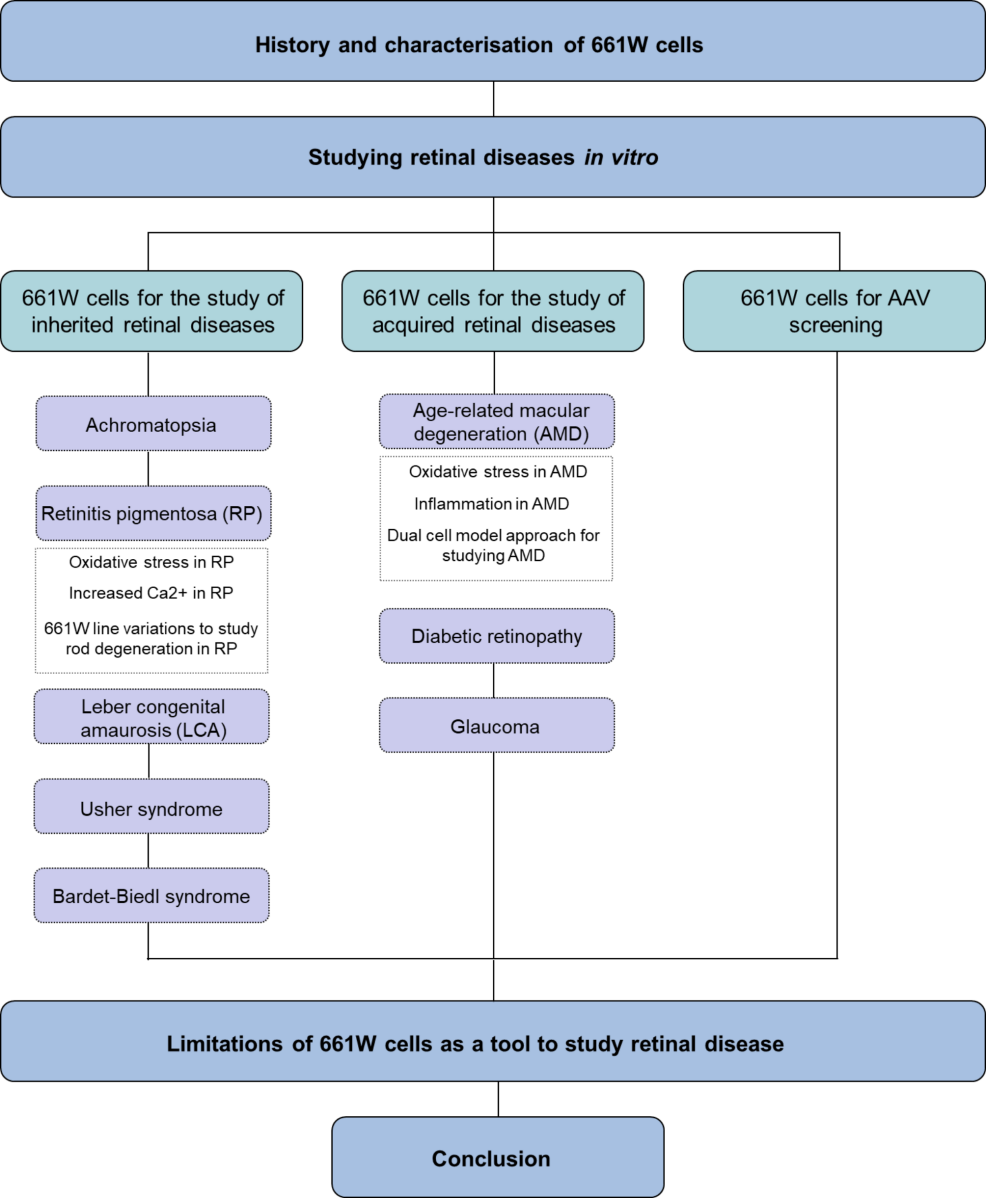
Flow chart illustrating the main topics of this review

## History and characterisation of 661W cells

The synthesis of the 661W cell line began in 1992 when Al-Ubaidi et al. were investigating the effects of expression of viral oncogenes in photoreceptors [4]. Previously, Al-Ubaidi et al. (1992) demonstrated that expression of the simian virus 40 (SV40) large tumour antigen (T antigen) driven by the rhodopsin promoter led to rod photoreceptor cell death rather than tumorigenesis [6]. As a result, the lab group investigated whether using the interphotoreceptor retinoid-binding protein (IRBP) promoter, which precedes the expression of rhodopsin during development as well as being expressed in both rods and cones, to drive the expression of SV40 T antigen would induce tumorigenesis in photoreceptors [4]. This led to early bilateral retinal and brain tumours being formed in transgenic mice. These transgenic mice were also crossbred with the retinal degeneration 1 (*rd1*) mouse that allowed identification of tumours forming, not only in rod photoreceptors, but in cones as well, as *rd1* mice exhibit early rod degeneration. Thus, the 661W cell line was isolated from these retinal tumours [4].

Although originally immortalized in 1992, the 661W cell line was not fully characterised until 2004 by the Al-Ubaidi group [5]. In culture, these cells adhere to the growth surface and initially exhibit an equivalent cytoplasm and nuclear size. Once attached, the cytoplasm increases in size and cells start to exhibit an elongated and spindle-shaped appearance. 661W cells form monolayers with a tendency to cluster, which facilitates the formation of cell-cell contacts, similar to those seen in retinal tissue [5]. The 661W cell lineage is known to proliferate rapidly and have a doubling rate of approximately 24 h as used commonly in cultured mediums like DMEM supplemented by bovine serums, and growth factors such as epidermal growth factor (EGF) or fibroblast growth factor (FGF) can enhance proliferation [7].

Interestingly, their morphology is not representative of photoreceptors as they lack the typical outer segments present in rods and cones [5]. Cellular and molecular analyses revealed that 661W cells more closely resemble cone photoreceptors in their physiological characteristics and gene expression pattern than rod photoreceptors. They express cone opsins, transducin and arrestin, proteins involved in the cone phototransduction cascade, but do not express rhodopsin or rod arrestin. Due to the expression of cone-specific genes, the 661W cell line can serve as a useful tool for investigating cone disease mechanism and treatment. Furthermore, upon exposure to light, 661W cells undergo changes in cyclic guanosine monophosphate (cGMP), and other signalling pathways typical of photoreceptor activity [8]. This light-induced response allows 661W cells to be used a model to study the molecular mechanisms underlying phototransduction and photoreceptor health. However, research has not yet shown that 661W cells respond to different wavelengths of light in a manner comparable to native photoreceptors. Nonetheless, research has utilised 661W cells to investigate how light exposure affects cellular processes, such as gene expression, protein phosphorylation, and cell survival, providing valuable insights into normal visual function and the pathophysiology of retinal disease [8–12]. As 661W cells lack the polarized morphology of photoreceptors, they are less suitable for studies involving light-mediated processes involving trafficking of typical outer segment proteins.

More recently, 661W cells have been characterised for the study of retinal ciliopathies. In a comprehensive study in 2019, Wheway et al. performed whole transcriptome sequencing of 661W cells to reveal the expression of numerous cilia-associated genes [13]. High-resolution imaging techniques confirmed the presence of distinct cilia structures and their related proteins [13]. Additionally, the knockdown of the cilia gene *lft88* effectively disrupted cilia formation in 661W cells [13]. Together, these studies have established 661W cells as a suitable model for investigating cone photoreceptor degeneration in retinal diseases.

## Studying retinal diseases in vitro

A variety of retinal cell lines have been developed to study retinal diseases, with immortalized cell lines being a convenient tool due to their ease of culture and reproducibility. Apart from 661W cells, other cell lines with photoreceptor similarities include the retinoblastoma Y-79 [14] and WERI-Rb [15] lines, and the MU-PH1 line [16]. The Y-79 cell line is a human-derived line that has been shown to express both rod an cone photoreceptor genes, but has a greater expression level of rod-specific genes [17, 18]. Expression of rod-specific genes has also been shown to be modulated by light, reflecting gene expression changes in rods [18]. As the Y-79 cell line is human-derived, it has more relevance in studying human retinal diseases over 661W cells which are murine-derived. However, the Y-79 line has been more commonly used in retinoblastoma research rather than as a model for studying photoreceptors. When treated with thyroid hormone, the WERI-Rb line expresses L and M opsin genes, though lack expression of other photoreceptor genes [19, 20], limiting its use in photoreceptor research. MU-PH1 cells are more reflective of progenitor cells as they have both Müller glia and photoreceptor characteristics [16]. They express both rod and cone photoreceptor genes, as well as melanopsin which is a gene specific to intrinsically photosensitive RGCs. The cell line was more recently developed compared to other photoreceptor lines [16], and as such there is limited research using this line. With these factors taken into consideration, the 661W cell line currently offers the most well-studied immortalized cell model for photoreceptor cells.

Retinal degeneration can also encompass the degeneration of the retinal pigment epithelial (RPE) cells, such as in age-related macular degeneration (AMD) [21]. The ARPE-19 cell line is the most commonly used model for studying RPE degeneration in retinal diseases [22], and can be investigated in parallel with 661W cells to model the RPE and photoreceptor degeneration seen in AMD patients. Retinal ganglion cells (RGCs) are the primary cells affected in optic neuropathies, and previous in vitro studies used the RGC-5 cell line to model these diseases [23]. Controversy surrounded the use of RGC-5 after conflicting experimental results prompted an in depth characterisation of the line, revealing that the RGC-5 line does not appropriately represent RGCs, with some journals now refusing to publish studies using this line [24]. The original paper characterizing the RGC-5 line has since been retracted [23]. These findings underscore the importance of fully characterizing cell lines before employing them to study diseases. Cell lines for other retinal cells include MIO-M1 cells as a model for Müller glia [25], and MG5 as a model for microglia [26].

Primary retinal cultures, a combination of cells isolated directly from the retina, provide the most physiologically relevant cell-based model for studying retinal function and degeneration [27]. These cultures retain key structural and functional properties, making them ideal for understanding disease mechanisms across retinal cell types and testing therapeutic interventions. To model different aspects of retinal degeneration, studies have exposed primary retinal cell cultures to conditions such as light induced-damage [28], hyperglycemia [29], and oxidative stress [30]. Neuroprotective treatments have been tested for photoreceptor degeneration in cell culture such as minocycline (an antibiotic) [30] and crocin (a painkiller) [28], amongst others. However, primary retinal cell cultures require additional supplements, neurotrophic factors and substrates to be maintained [31, 32], adding to the cost of experiments and inducing possible physiological changes that are not representative of the typical retinal environment. Culturing isolated photoreceptor cells, as opposed to retinal cell cultures consisting of a variety of cells, has proven difficult as they often lose their inner and outer segments during the isolation process, likely contributing to their 1–2 day limited lifespan [31]. Unlike primary photoreceptor cultures, which have a limited lifespan and require continuous harvesting from animals [31], 661W cells provide a consistent and reliable supply of photoreceptor cells for experiments.

Retinal organoids, which are retinal-like tissue derived from human pluripotent stem cells (hPSCs), provide a comparable tissue culture model to the retina as it mimics the cellular architecture and molecular function of the human retina [33]. The laminated 3-D structure of retinal organoids allows for cellular interactions and microenvironmental conditions that recapitulate the retina [33]. Since retinal organoids are often derived from hPSCs, they provide a human-specific model, limiting inter-species differences that arise from animal cell lines. Furthermore, retinal organoids derived from patients harbouring disease mutations exhibit similar pathophysiology, providing a platform to study morphological and molecular changes associated with disease-causing mutations [7, 34, 35]. However, retinal organoids are costly, difficult to maintain, and have a complex and timely differentiation process [36], as differentiation typically takes around 100 days to form mature and functional photoreceptors [37]. This makes retinal organoid impractical for wide drug screening experiments, though valuable for patient-tailored interventions.

While primary retinal cell cultures and retinal organoids provide the most physiologically relevant models, their limitations in scalability and maintenance make immortalized cell lines like 661W cells a valuable alternative for preliminary investigations into retinal diseases. Since their development, 661W cells have significantly advanced research in a variety of retinal diseases, with the studies discussed in this review outlined in Table 1.

**Table 1:** Studies using the 661W cell line discussed within this review

Application	Disease modelling	Paper	Findings
Inherited retinal diseases			
Achromatopsia	Exogenous expression of human mutations	Zheng et al. [38]	R410W mutation in *CNGA3* leads to hyperactive CNG channel
	Exogenous expression of human mutations	Liu et al. [39]	F525N or T383fsX mutations in *CNGB3* leads to hyperactive CNG channels and increased cytotoxicity
	Exogenous expression of human mutations	Duricka et al. [40]	R563H or Q655X mutations in *CNGA3* leads to ER stress
Retinitis pigmentosa	Oxidative stress – H_2_O_2_ induced	Fabiani et al. [41]	Inhibiting ceramide or increasing sphingosine-1-phosphate activity reduced cell death
	Oxidative stress – H_2_O_2_ induced	Tahia et al. [42]	Inhibiting ceramide biosynthesis reduced cell death and by modulating antioxidant, apoptotic and sphingolipid pathways
	Oxidative stress – H_2_O_2_ induced	Leyk et at. [43]	Tubastatin A (HDAC6 inhibitor) improved cell viability and maintained healthy cell biology
	Oxidative stress – Light damage	Zhu et al. [44]	Inhibition of PKM2 reduced cell death and oxidative stress
	Increased Ca^2+^	Perron et al. [45]	SAHA (broad HDAC inhibitor) reduced cell death whilst Tubastatin A did not
	Increased Ca^2+^	Arroba et al. [46]	IGF-I reduced cell death, decreased calpain-2 activation, increased calpastatin levels
	Increased Ca^2+^	Lin et al. [47]	S6K1 is critical for cell survival
	Increased Ca^2+^	Luodan et al. [48]	Metformin reduced cell death
A model for rod photoreceptors	Exogenous expression of human mutations	Liu et al. [49]	P23H, R135L, or G188R mutations in *RHO* lead to energy failure and OXPHOS deficiency
	Phosphodiesterase 6 inhibition	Huang et al. [50]	Using zaprinast to inhibit PDE6, cells exhibited increased cGMP and Ca^2+^ levels, and activation of PKG and calpains, modelling rod degeneration in retinitis pigmentosa
Leber congenital amaurosis	Exogenous expression of healthy gene	Tang et al. [51]	Exogenous *RPE65* expression in 661W cells supports function
	Exogenous expression of human mutation	Minegeshi et al. [52]	T400P or R516H mutations in *CCT2* induce a partial, and not complete, defect in CCT machinery
Usher syndrome	Exogenous expression of human mutation	Panagiotopoulos et al. [53]	c.254-649T > G mutation in *CLRN1* can be corrected in 661W cells using antisense oligonucleotide therapy
Bardet-biedl syndrome	Overexpression of protein	Zhang et al. [54]	Overexpression of Rnf217 protein leads to downregulation of Bardet-biedl syndrome genes
Acquired retinal diseases			
Age-related macular degeneration	Oxidative stress – atRAL-loaded cells	He et al. [55]	atRAL accumulation impairs ER function, eIF2a activation, and cell death via JNK signalling dependent apoptosis and GSDME pyroptosis
	Oxidative stress – atRAL-loaded cells	Yang et al. [56]	Crocin improved cell viability, amerliorated oxidative stress and mitochondrial damage, and reduced apoptosis, pyroptosis and ferroptosis
	Oxidative stress – atRAL-loaded cells	Ortega et al. [57]	Flavonoids quercetin and myricetin improved cell viability increased expression of M- and S-cone opsin genes, and promoted pro-survival pathways
	Oxidative stress - SO_2_-induced	Du et al. [58]	Inhibition of AAT1 inhibited SO2 synthesis, partially mimicking H_2_O_2_-induced apoptosis
	Oxidative stress – H_2_O_2_-induced	Dong et al. [59]	ERK1/2 and STAT3 signalling was increased after H_2_O_2_-induced oxidative stress. Inhibiting either ERK1/2 or STAT3 exacerbated cell death.
	Oxidative stress – H_2_O_2_-induced	Sánchez-Bretaño et al. [60]	Melatonin partially ameliorated cell death by activating melatonin receptors
	Oxidative stress – H_2_O_2_-induced	Baba et al. [61]	661W cells posses a circadian clock, and protects against oxidative stress via modulation of glutathione peroxidase activity
	Oxidative stress – H_2_O_2_-induced	Ortega et al. [57]	Flavonoids quercetin and myricetin improved cell viability increased expression of M- and S-cone opsin genes, and promoted pro-survival pathways
	Oxidative stress – light damage	Chen et al. ([8])	Nrf2 protects cells by activating the antioxidant response element
	Oxidative stress – light damage	Natoli et al. [10]	Pyruvate protects against oxidative damage
	Oxidative stress – light damage	Mandal et al. [62]	Curcumin protects against oxidative damage
	Oxidative stress – light damage	Lin et al. ([9])	Astaxanthin protects against oxidative damage
	Oxidative stress – tBHP-induced	Ma et al. [63]	Elamipretide (SS31) protects against oxidative damage
	Oxidative stress – High glucose	Lai et al. [64]	Astaxanthin protects against oxidative damage
	Inflammation	Shi et al. [65]	C5b-9 can sensitise 661W cells to certain apoptotic and necroptotic pathways,
	Inflammation	Lee et al. [66]	VEGF-treatment in 661W cells induced the expression of inflammatory proteins. Flavonoid quercetin suppressed inflammatory molecules, inhibited the angiogenic response, and inactivated of the NF-kB pathway via the inhibition of MAPK and AKT phosphorylation
	Inflammation	Kamoshita et al. [67]	AICAR reduced inflammatory cytokine *Tnf-a* mRNA levels, and increasing the mRNA levels of *Pgc1-a*, a mitochondrial biogenesis regulator.
		Schnichels et al. [68]	anti-VEGF drug aflibercept had no toxic effects on 661W cells
	Iron overload	Huang et al. [69]	Induced senescence-like changes, impaired cell proliferation, mitochondrial dysfunction, and apoptotic cell death. Cells exhibited activation of MAPK and its downstream molecules
Diabetic retinopathy	High glucose	Lam et al. [70]	Proteomic analysis identified an increase in apoptosis and ROS
	High glucose	Taki et al. [71]	Impairment of autophagy causes superoxide formation and caspase activation
	High glucose	Arroba et al. [72]	Somatostatin moderately promotes cell survival
	High glucose	Lv et al. [73]	Sulforaphane delays cell death
	Advanced glycation end product-induced	Song et al. [74]	Addition of AGEs mimicked diabetic retinopathy conditions
Glaucoma	Staurosporine for 661W cell differentiation	Sayyad et al. [75]	Induces 661W cell differentiation into retinal ganglion cells
	Pressure-induced	Somvanshi et al. [76]	Cannabinol decreased cell death
	Light-induced	Imamura et al. [77]	Rimonabant, a selective cannabinoid receptor agonist, decreased cell death
	Light-induced	Imamura et al. [78]	HU-308, an agonist of cannabinoid receptor type 2, reduced cell death
	Expression of mutated gene	Zhu et al. [79]	Expression of *MYOC* mutation led to decreased autophagy activity, and increased mitochondrial dysfunction and oxidative stress
	Expression of mutated gene	Sayyad et al. [75]	Expression of *OPTN* mutation decreased cell viability
	Overexpression of gene	Chen et al. [80]	Overexpression of *OPTN* led to autophagy. Treatment with acteoside reduced autophagy
AAV screening		Ryals et al. [81]	scAAV1 and scAAV2 were more efficient than scAAV5 and scAAV8 vectors at transducing 661W cells. Increasing the number of Y-F capsid mutations increased transduction efficiency. A sextuple mutant scAAV2 showed a nine-fold increase in transduction efficiency to unmodified scAAV2
		Kay et al. [82]	Screened various capsid-mutated AAV vectors and found scAAV2 (quadY-F-T-V) to be the most efficient
		Boye et al. [83]	Preincubation of AAV2-smCBA-mCherry with Healon for varying durations showed up to a three-fold increase in transduction efficiency after one hour
		Böhm et al. [84]	Testing CRISPR-Cas9 plasmid transfection fast-tracked the feasibility testing of this system before in vivo applications

## 661W cells for the study of inherited retinal diseases

### Achromatopsia

Given that 661W cells are characterised as a cone-like cell line, they are particularly well-suited for investigating cone dystrophies. Achromatopsia is a cone dystrophy typically caused by mutations in genes involved in cone phototransduction, resulting in dysfunction and/or degeneration of cone photoreceptors [38]. This condition results in severe visual impairments characterised by significantly reduced daylight vision and visual acuity, as well as loss of colour vision. Achromatopsia affects 1:30,000 people globally, with currently no available treatments for this condition [39, 40]. The most prevalent causes of achromatopsia are mutations in the alpha and beta subunits of the cyclic nucleotide gated channel (*CNGA3* and *CNGB3*) genes, accounting for approximately 25–30% and 40–50% of achromatopsia cases, respectively [38]. Prevalence of these disease genes is population dependent, and *CNGA3* mutations can account for as high as 84% and 80% of all achromatopsia cases in Israeli/Palestinian [41] and Chinese populations [42], respectively. Investigating these gene mutations is therefore of high relevance in understanding disease mechanisms and developing treatments for the majority of achromatopsia patients.

Both the *CNGA3* and *CNGB3* disease genes are being investigated for viral-based gene therapy in clinical trials. The *CNGA3* gene therapy treatment has shown only minor improvements to patient’s visual acuity and colour discrimination (NCT02610582) [43]. Similarly, the *CNGB3* clinical trial also reported minor improvements to colour vision and photoaversion in some patients, though 21 of 23 treated patients reported increase in vision-related quality-of-life (NCT03001310) [44]. However, electroretinogram responses remained unmeasurable in all patients within both clinical trials [43, 44]. Further pre-clinical studies are essential for improving the efficacy of these gene therapy treatments, possibly with subtle variations to the viral engineering of the gene therapies. For example, the viral construct used in the *CNGB3* trials employed the human cone arrestin promoter to drive expression, though its use was superseded by the preferred L-opsin promoter (PR1.7) in more recent pre-clinical testing [45]. 661W cells provide an ideal model for fast-tracking gene therapy safety and transfection efficacy testing and could be a potential avenue for testing multiple gene therapies. It is important to note that 661W research provides just a foundation for screening of gene therapies, and once a suitable therapy has been identified, its effects in an appropriate animal model would need to be tested before progressing to clinical trials.

661W cells can also aid in identifying the pathophysiological mechanisms of cone degeneration in achromatopsia disease genes. However, it has been previously reported that 661W cells do not express endogenous *CNGA3* and have only minimal expression of *CNGB3* [46], requiring exogenous expression of these genes for relevant studies. 661W cells expressing different human *CNGA3* and *CNGB3* mutations provided valuable insight into disease mechanisms and revealed cytotoxicity dependent on increased intracellular calcium (Ca^2+^) and cyclic guanosine monophosphate (cGMP) [47–49], as well as increased unfolded protein response (UPR) markers [49]. Additionally, blocking of cyclic nucleotide gated (CNG) channels or removal of extracellular Ca^2+^ rescued 661W cell viability, suggesting high intracellular Ca^2+^ as being a primary contributor in CNG-associated cone degeneration [48]. These pathophysiological mechanisms of *CNGA3* and *CNGB3* mutations are mirrored in animal studies [50–52], giving credence to the use of 661W cells in cone degeneration study, even if the disease-associated gene is not endogenously expressed.

Research is limited for the remaining achromatopsia-associated disease genes which include: *GNAT2*, *PDE6C*, *PDE6H*, and *ATF6*. Although each of these genes account for less than 5% of achromatopsia cases globally [38], certain populations show significantly higher incidence rates, such as Korean populations exhibiting up to 38% of patients affected with *PDE6C*-associated achromatopsia [53]. Their low global incident rate is most likely the reason why research efforts have been focused more towards *CNGA3* and *CNGB3* genes instead. Although not yet explored, 661W cells may be an ideal, cost-efficient model to study these disease-associated genes. Expression of *GNAT2* [5, 54, 55], *PDE6H* [55], *ATF6* [56] have previously been confirmed in 661W cells, but reports were not able to confirm *PDE6C* expression [13, 54]. Future directions for the use of 661W cells may include functional knockdown of the achromatopsia genes that are endogenously expressed to elucidate the pathophysiological mechanisms of cone degeneration in vitro. Furthermore, transgenic expression of associated mutations in 661W cells may provide greater insight into patient specific mutations as was found in *CNGA3* and *CNGB3* mutation studies [47–49].

### Retinitis pigmentosa (RP)

Retinitis pigmentosa (RP) is a rod-cone dystrophy and stands as the most prominent form of inherited retinal disease [57, 58]. In contrast to the low incidence rates of achromatopsia, RP accounts for approximately 40% of IRDs, and affects 1:4000 people globally [57]. Mutations that cause RP typically occur in rod-specific genes, resulting in primary rod death, followed by a secondary, mutation-independent cone degeneration. As such, the disease initially manifests as night-blindness (nyctalopia), and as the disease advances, patients experience a gradual constriction of the visual field (tunnel vision) which can progress to complete blindness [59]. Although cones are not genetically affected, understanding the mechanisms by which they degenerate, and identifying therapeutic targets is crucial, given our reliance on cone-mediated vision in daily life. The underlying mechanisms of cone degeneration in RP remain unclear, however, they are thought to involve processes such as oxidative stress, Ca^2+^ overload, and dysregulation of key metabolic pathways, all of which have been studied in 661W cells.

#### Oxidative stress in RP

Oxidative stress is a major factor involved in the pathophysiology of RP, with an imbalance between reactive oxygen species (ROS) production and antioxidant defences mechanisms leading to photoreceptor damage and death [60]. In 661W cells, oxidative stress is commonly induced through methods such as H_2_O_2_-treatment, and light damage, providing valuable insights into potential therapeutic targets and neuroprotective therapies in RP. For instance, under oxidative stress, various strategies have shown success in rescuing 661W cells, including promoting pro-survival pathways [61, 62], inhibiting pro-apoptotic pathways [63], and inhibiting histone modifiers [64].

Apoptosis is a well-established death process occurring in RP, and has been shown to be a major cell death pathway in 661W cells exposed to oxidative stress [65]. Ceramide (CER) and sphingosine-1-phosphate (S1P) are sphingolipids with opposing actions: CER promotes apoptosis, while S1P promotes cell survival [66]. In 661W cells under H_2_O_2_-induced oxidative stress, pre-treatment with an inhibitor of *de novo* CER synthesis demonstrated protective effects [61]. Similarly, pharmacologically increasing S1P levels protected cells by impairing apoptosis and promoting pro-survival responses. Another study that inhibited ceramide biosynthesis with L-cycloserine showed 661W cells were protected from oxidative-stress mediated cell death through modulating antioxidant, apoptotic and sphingolipids pathway genes [62]. Studies have also used 661W cells to investigate molecular changes during oxidative stress and identify potential therapeutic targets. One such study investigated the role of pyruvate kinase 2 (PKM2), which is expressed in photoreceptors and has been associated with oxidative stress [63]. In response to light damage, 661W cells featured increased PKM2 expression, oxidative stress, and apoptosis. Pharmacological PKM2 inhibition improved these parameters, suggesting PKM2 may be a promising therapeutic target.

Another avenue of research has focused on histone deacetylase (HDAC) activity, which has been shown to be dysregulated in several IRD models, including RP [67]. HDAC inhibitors have shown promise in protecting 661W cells from oxidative stress [64], and have emerged as a promising neuroprotective strategy. A study by Carullo et al. (2024) developed a variety of HDAC inhibitors and selected the most suitable inhibitors to test their mechanism of action and ability to combat oxidative stress in both 661W and ARPE-19 cells. In 661W and ARPE-19 cells under H_2_O_2_-induced oxidative stress, pre-incubation with different HDAC inhibitors saw significant improvements in cell viability and maintained healthy cellular morphology compared to H_2_O_2_ treatment alone [64]. Their developed HDAC inhibitor, repistat, was chosen for testing in the dye^ucd6^ zebrafish and *rd10* mouse model of inherited retinal disease and demonstrated the ability to alleviate cone degeneration. This thorough study exemplifies the importance of using multiple models to validate a treatment, with parallel testing of 661W and ARPE-19 cells serving as a preliminary screening platform to assess drug efficacy before progressing to more complex in vivo models.

#### Increased Ca^2+^ in RP

Alongside oxidative stress, increased Ca^2+^ levels are a common feature in many RP models and contribute significantly to photoreceptor degeneration. Elevated Ca^2+^ levels have been widely studied in 661W cells and have been used to assess various drugs for neuroprotective effects. Suberoylanilide hydroxamic acid (SAHA) [68], insulin like-growth factor-1 (IGF-1) [69], and metformin [70] have all demonstrated protective effects, and have partially recovered viability of 661W cells under Ca^2+^ overload.

In 661W cells stressed with the calcium ionophore A23187 to increase intracellular Ca^2+^, pre-treatment with SAHA (a broad-acting pan-HDAC inhibitor) improved cell survival and redox capacity [68]. These protective effects were also observed in retinal explants from the *rd1* mouse model of RP, where SAHA treatment resulted in a two-fold increase in photoreceptor numbers at the highest concentration tested [68]. Interestingly, in the same study, Tubastatin A, which was previously shown to be effective against oxidative stress in 661W cells [64], was unable to mitigate the effects of Ca^2+^ overload, highlighting the importance of testing drug efficacy under different disease-related cellular conditions.

In a notable study by Arroba et al. (2009), IGF-1 was shown to protect the 661W cells from Ca^2+^-induced apoptosis, reduce calpain-2 activation, and maintain levels of calpastatin via the AKT-CREB pathway [69]. The therapeutic effects of IGF-1 were further validated in wildtype retinal explants under Ca^2+^ stress, and explants from the *rd1* mouse model of RP. In both models, IGF-1 reduced photoreceptor apoptosis, decreased calpain-2 activation, and increased calpastatin levels, mirroring the molecular findings from 661W cells. These findings underscore the value of 661W cell research in identifying potential molecular mechanisms associated with therapeutic compounds before these therapies are transitioned into more complex models.

Another critical pathway involved in RP is the mammalian target of rapamycin (mTOR) signalling pathway, a key regulator of cell metabolism, growth, and survival. Aberrant activation of mTOR signalling in some RP models has made it an attractive target for therapeutic intervention [71, 72]. Research by Lin et al. (2018) showed that ribosomal S6 kinase 1 (S6K1), a downstream effector of mTOR, was critical for 661W cell survival [73]. This was further corroborated in vivo in the *rd10* mouse model of RP, where overexpression of S6K1 promoted both rod and cone survival and function [73]. Additionally, metformin, a widely available anti-diabetic drug and inhibitor of mTOR complex 1 (mTORC1) has also shown therapeutic potential. Intravitreal injections of metformin in the *rd1* mouse delayed visual impairment and reduced photoreceptor apoptosis [70]. 661W experiments supported metformin’s protective effects against Ca^2+^-induced apoptosis, highlighting the therapeutic potential of targeting mTOR and its downstream effectors in RP treatment [70].

#### 661W line variations to study rod degeneration in RP

Manipulating the 661W cell environment to model secondary cone degeneration has provided valuable insights into molecular mechanisms, therapeutic targets, and potential treatments for RP. However, as RP is characterised by primary rod degeneration, the limitations of 661W cells as cone-like cells must be acknowledged. A recent study by Liu et al. (2022) overexpressed rhodopsin (RHO) in 661W cells to examine the effects of autosomal dominant RHO mutations on energy metabolism in photoreceptors [74]. Wild-type (WT), RHO, and three mutants (P23H, R135L, and G188R) were overexpressed in 661W cells, and they found that in RHO overexpressed cells, energy failure may also be one of the early events involved in primary rod death. Additionally, the authors found RHO overexpression in the WT and P23H groups led to OXPHOS deficiency, triggering AMPK activation and metabolic reprogramming towards increased aerobic glycolysis. However, energy failure and cell injury were more severe in the R135L and G188R mutants, potentially from impaired metabolic reprogramming [74]. These results generally correlate with the clinical severity of these mutations (R135L > G188R > P23H). While 661W cells cannot fully replicate in vivo photoreceptors, this RHO overexpression model may be a useful in vitro tool to investigate the mechanisms underlying the heterogenous phenotypes of RHO mutations.

A novel 661W-A11 line has also been recently developed by Huang et al. (2021) which expresses rod-specific genes to better represent rods in vitro [54]. During retinal development, photoreceptor precursor cells are inherently determined to differentiate into cone cells, unless expression of neural retina-specific leucine (*Nrl*) is present [75]. 661W-A11 cells transfected with *Nrl* showed increased expression of rod genes, though maintained similar expression levels of cone genes to the original 661W line [54]. However, morphologically they differed, with 661W-A11 being more elongated and having a slower replication rate. Using zaprinast to inhibit phosphodiesterase 6 (PDE6) activity in 661W-A11 has shown promise in mimicking the pathophysiology of RP rods. These cells exhibited increased cyclic-guanosine mono phosphate (cGMP) and Ca^2+^ levels, along with activation of protein kinase G (PKG) and calpains [54]. Contrastingly, some studies suggest the original 661W line is insensitive to zaprinast treatment [76, 77]. While the full potential of the 661W-A11 cell line remains to be explored, its recent development represents a significant advancement in facilitating future RP research.

### Leber congenital amaurosis

Leber congenital amaurosis (LCA) is a rare disease considered to be a severe and early onset form of retinitis pigmentosa due to the similar clinical features [78]. Symptoms are present from birth or manifest in early infancy. There have been over 20 disease-associated genes identified, some of which are also associated with the later onset retinitis pigmentosa [40]. However, the use of 661W cells to study these 20 LCA genes is limited.

The *RPE65* gene is arguably the most well-known LCA-related gene, as it was the first to have an FDA-approved adeno-associated viral vector gene therapy Luxturna^®^ (voretigene neparvovec-rzyl) [3]. Mutations to the *RPE65* gene, leading to defective RPE cells, is one of the common causes of LCA, though incidence rates vary largely within populations [79]. After phase 1 clinical trials of Luxturna^®^, Tang et al. (2011) identified *RPE65* to also be expressed in human cones [80]. They further demonstrated in 661W cells that exogenous expression of *RPE65* supports cone function by promoting photopigment regeneration, expanding our understanding of the therapeutic impact of Luxturna^®^. These retrospective findings of developed therapies are important in guiding development of future therapies, particularly in 661W cells, as it expands their experimental potential for use in studying mutations in genes not endogenous to 661W cells.

Another study explored mutation-specific defects in the *CCT2* gene identified within a family exhibiting LCA symptoms [81]. The study attempted to generate retinal organoid cultures from the patient-derived induced pluripotent stem cells, but were unsuccessful due to decreased proliferative activity in the organoids. Knockdown of the *CCT2* gene in 661W cells also found decreased proliferation. Using a viral vector, overexpression of wildtype *CCT2* significantly rescued cell proliferation, while overexpression of its mutated form had no rescue effect [81]. Though the same experimental design may have been conducted in retinal organoids, the ease of use and maintenance of 661W cells allowed for easier collection of results, highlighting their value in functional studies of gene mutations.

### Usher syndrome

Usher syndrome is a rare ciliopathy that also displays similar clinical presentations to retinitis pigmentosa, however, patients with Usher syndrome exhibit sensorineural hearing loss [82]. It is the most common condition affecting both vision and hearing, with varying degrees of severity depending on the mutation [82, 83]. Usher syndrome is inherited in an autosomal recessive manner and is categorised into three primary types, 1, 2, 3, each distinguished by the onset and severity of hearing loss and rate of vision decline [82]. To date, there have been 15 genes associated with this condition [40]. Although animal models sufficiently represent the hearing loss aspect of Usher syndrome, validation of the retinal degeneration that mimics the human presentation in mouse models are still lacking [84]. Recent advances in genome editing technologies, such as CRISPR-Cas9, have made it possible to develop a genetically modified primate model of Usher syndrome using the *MYO7A* gene [85]. However, generating these in vivo models requires a substantial amount of time and validation.

Despite Wheway et al. (2019) having established 661W cells as a suitable model for studying ciliopathies [13], their use for investigating Usher syndrome remains underexplored. One study successfully introduced an Usher-causing mutation in the *CLRN1* gene into 661W cells, and further demonstrated that the mutation could be corrected using an antisense oligonucleotide approach [86]. However, studies involving other Usher-associated genes in 661W cells are limited, highlighting a significant opportunity for future research within this area.

### Bardet-Biedl syndrome

Bardet-biedl syndrome is another rare ciliopathy that is characterised by an expansive range of debilitating symptoms that affects many body systems, including cognitive impairment, renal dysfunction, obesity, heart defects, as well as rod-cone dystrophy [87]. The retinal degeneration aspect of Bardet-biedl syndrome is often progressive, with symptoms becoming apparent by 7 or 8 years of age and many patients becoming legally blind by their mid-teens [88]. Additional ocular dysfunctions associated with Bardet-biedl syndrome include strabismus, cataract, and glaucoma [89]. Although 24 disease-associated genes have been identified [40], approximately 20–30% of patients have unknown genetic causes [90]. This gap in knowledge complicates diagnosis and treatment for a substantial subset of Bardet-biedl syndrome patients.

The use of 661W could potentially expedite the identification process of disease genes for the remaining patients. A novel study by Zhang et al. (2020) demonstrated that the mature microRNA miR-183 is indispensable for vision, with its ablation resulting in the downregulation of Bardet-biedl syndrome (BBS) genes [91]. Given that microRNAs negatively regulate the expression of numerous genes, the study sought to identify the mechanism by which miR-183 downregulation led to retinal dysfunction. The overexpression of Rnf217 protein, a direct target of miR-183, in 661W cells confirmed the subsequent downregulation of BBS genes. Although neither *miR-183* or *RNF217* have thus far been identified as disease genes in Bardet-biedl patients, *miR-183* and its respective targets may be of interest when conducting genetic screening of patients. The study by Zhang et al. has also paved the way for using 661W cells to further investigate retinal degeneration in Bardet-biedl syndrome.

## 661W cells for the study of acquired retinal diseases

### Age-related macular degeneration (AMD)

Age-related macular degeneration is a progressive retinal disease characterised by the degeneration of cells in the macula, the central portion of the retina responsible for central vision. The macula contains a cone-rich area called the fovea, and the loss of cone photoreceptors in this region causes major visual impairment [59]. The two primary types of the AMD are neovascular and geographic, differentiated by the presence of neovascularisation and leaking of fluid from the retinal vasculature in neovascular AMD. Geographic AMD may also develop into neovascular AMD in patients at advanced stages of the disease. Treatments for neovascular AMD are aimed at decreasing vasculature atrophy via anti-VEGF injections and laser treatment; however, no treatments currently exist for geographic AMD. Furthermore, the underlying mechanisms that contribute to AMD pathogenesis have been widely studied but are still not fully understood.

661W cells have been widely used to study the molecular mechanisms of cone degeneration within the context of AMD, with enough research available to warrant its own dedicated review. Similar to retinitis pigmentosa, oxidative stress is a well-established contributor to AMD and can be easily replicated in vitro [9, 10, 92]. However, this overlap in disease modelling brings into question whether 661W cells could accurately represent disease processes in two vastly different diseases.

#### Oxidative stress in AMD

Several studies have examined the molecular mechanisms associated with oxidative stress within the AMD retina, which include (but are not limited to) the role of all-trans-retinal (atRAL) accumulation, endogenous SO2, and ERK1/2 and STAT3 signalling activation [93, 94]. The accumulation of atRAL in photoreceptors has been closely associated with geographic AMD. A recent study by He et al. (2023) utilised both atRAL-loaded 661W cells and mouse models to investigate the molecular mechanisms of atRAL mediated cell death. The authors propose that atRAL accumulation impairs ER function, partially through oxidative stress, which leads to eukaryotic translation initiation factor 2α (eIF2α) activation, promoting retinal degeneration via c-Jun N-terminal kinase (JNK) signalling-dependent apoptosis and gasdermin E (GSDME) mediated pyroptosis [93]. In atRAL-loaded 661W cells, crocin, a natural carotenoid with antioxidant and anti-inflammatory properties used for pain relief, was found to improve cell viability, ameliorate oxidative stress and mitochondrial damage, and reduce apoptosis, pyroptosis and ferroptosis [94]. Interestingly, in light-damaged mice, crocin treatment improved visual function and retinal integrity compared to untreated controls, highlighting its therapeutic potential [95].

Given the central role oxidative stress plays in AMD pathogenesis, it is crucial to understand how it can affect other cellular pathways. In this context, 661W cells treated with H_2_O_2_ exhibited significantly reduced endogenous sulphur dioxide (SO_2_) levels and aspartate aminotransferase 1 (AAT1) expression, thereby promoting apoptosis [96]. Inhibition of AAT1 inhibited SO_2_ synthesis, and partially mimicked H_2_O_2_-induced apoptosis seen in vitro. These findings indicated that the SO_2_/AAT1 pathway may be an important regulator of apoptosis, and that targeting this pathway may have therapeutic potential in AMD [96]. However, further investigations are necessary to clarify the specific mechanisms. In contrast to the pro-apoptotic SO_2_/AAT1 pathway, the activation of the ERK1/2 and STAT3 signalling pathways may serve as a protective response to oxidative stress. A study by Dong et al. (2012) found increasing activation of the ERK1/2 and STAT3 signalling pathways in response to increasing concentrations of H_2_O_2_ [97]. Pharmacologically inhibiting ERK1/2 and STAT3 separately further aggravated H_2_O_2_-induced cell death, highlighting their potential role in combating oxidative stress.

Like in RP, research in AMD has also explored administration of antioxidants to counteract the effects of oxidative stress. 661W cells were used to examine the neuroprotective potential of Nrf2, which protects cells via the activation of the antioxidant response element, increasing the expression of an array of antioxidant genes [9]. Other antioxidants tested in 661W, including pyruvate [11], elamipretide [98], curcumin [99] and astaxanthin [10, 92], have also shown protective effects. While studies using 661W cells highlight its efficacy against oxidative damage [10, 92], human trials involving oral astaxanthin combined with other antioxidants have only shown modest benefits for AMD patients. Although there were reports of disease stabilisation and minor improvements to vision, the full extent of antioxidant supplementation for improving vision in AMD patients remains unclear [100, 101].

Melatonin, commonly known for its role in promoting sleep, is a natural antioxidant that has been studied for its protective effects in AMD. Human studies suggest a reduction in melatonin AMD patients [102], and further research has indicated that daily oral supplementation of 3 mg melatonin may help delay disease progression [103]. These findings suggest melatonin may have therapeutic potential for AMD, and researchers have further investigated these protective mechanisms in 661W cells. In a study utilising H_2_O_2_-stressed 661W cells, melatonin partially ameliorated cell death by activating its receptors, rather than its direct antioxidant action [104]. Notably, H_2_O_2_ induced the gene expression of apoptotic markers *Fas*,* FasL*, and *caspase-3*, but treatment with melatonin or a melatonin receptor agonist partially restored expression to basal levels, suggesting receptor-mediated protection [104]. Melatonin is also an important regulator of circadian rhythm [103], and while evidence of a direct correlation between melatonin, oxidative stress and circadian rhythm in AMD is currently lacking, a study by Baba et al. (2022) found that 661W cells may possess a functional circadian clock that could be useful for exploring such a relationship [105]. Furthermore, they demonstrated that the 661W circadian clock may protect cells from oxidative stress via modulation of glutathione peroxidase activity.

#### Inflammation in AMD

In vitro studies have also been useful in elucidating some of the underlying mechanisms of retinal inflammation, another key contributor to AMD. Research in 661W cells has shown that inflammation in AMD involves several factors, including the complement system [106], VEGF [107, 108] and pro-inflammatory signalling pathways [109–111]. The complement system, while a crucial host defence, has also been implicated in the pathogenesis of AMD due to its inflammatory mediators [112, 113]. Many different proteins are involved in the complement system, however a study by Shi et al. (2015) used 661W cells to investigate the role of the terminal complement complex C5b-9 in modulating photoreceptor death [106]. They found that C5b-9 can sensitise 661W cells to certain apoptotic and necroptotic pathways, suggesting that complement activation in AMD may directly induce cone death, or sensitise the cones to other insults, although further in vivo research is required [106].

VEGF is another key factor in the pathogenesis of neovascular AMD and drives inflammation and proliferative vascularisation. Elevated VEGF levels are common amongst AMD patients, and anti-VEGF intravitreal injections are commonly used as treatment. 661W cells have been used to assess treatments that modulate VEGF. One study used various ocular cell lines, including 661W cells, to test the toxicity of the approved anti-VEGF drug aflibercept [108]. Over the 72-hour period investigated, aflibercept showed no toxic effects in vitro at clinically relevant concentrations, reinforcing its safety for use in AMD patients. Another study found that VEGF-treatment in 661W cells induced the expression of inflammatory proteins, but that the protective flavonoid quercetin suppressed inflammatory molecules, inhibited the angiogenic response in vitro, and inactivated of the NF-kB pathway via the inhibition of MAPK and AKT phosphorylation [107].

Other studies have utilised 661W cells to investigate pharmacological agents that may mitigate the inflammatory processes and offer potential therapeutic strategies. This includes a study by Ortega et al. (2021) which used both in vitro and in vivo experiments to investigate the therapeutic potential of the flavonoids quercetin and myricetin [109]. In H_2_O_2_ and atRAL stressed 661W cells, both compounds improved cell viability, increased expression of M- and S-cone opsin genes, and promoted pro-survival pathways. Similarly, a study by Kamoshita et al. (2016) used 661W cells and in vivo models to assess the underlying mechanisms and protective effects of AICAR, an AMPK activator, during inflammation [110]. 661W cells treated with AICAR activated AMPK, reducing inflammatory cytokine *Tnf-a* mRNA levels, and increasing the mRNA levels of *Pgc1-a*, a mitochondrial biogenesis regulator.

#### Dual cell model approach for studying AMD

While 661W cells are a valuable tool to study photoreceptor degeneration, AMD is a multifactorial disease, in which a major hallmark is the degeneration of the RPE cells. Accordingly, a large amount of research has also focused on RPE-cell models, such as the ARPE-19 line, to better understand this crucial aspect of the disease. A limitation of inducing acute oxidative stress in 661W cells, is that it cannot mimic the gradual progression of AMD which is also accompanied by the accumulation of iron ions [114]. To address this, Huang et al. in 2024 generated chronic injury models in ARPE-19 and 661W cells by exposing them to iron ion overload over time [115]. This led to senescence-like changes in both cell lines, impaired cell proliferation, mitochondrial dysfunction, and apoptotic cell death. Both cell models exhibited activation of MAPK and its downstream molecules, and when intravitreally injected in mice, resulted in geographic AMD-like lesions and reduced visual function. This newly identified method of modelling a chronic AMD-like environment in 661W cells provides an alternative to the previously used acute oxidative stress model. This research helps support previous findings of the involvement of iron accumulation in AMD pathogenesis, while concurrently demonstrating that evaluating both ARPE-19 and 661W cells produced the same experimental outcome [115]. Future research on AMD disease mechanisms would benefit from incorporating both ARPE-19 and 661W cells, as this dual approach allows for a more comprehensive assessment of the disease, capturing both RPE and cone photoreceptor dynamics, which together offer a fuller model of retinal degeneration involved in AMD.

Overall, it is clear that 661W cells are a valuable model for understanding the mechanisms underlying AMD and identifying therapeutic targets. While some of the promising in vitro results have been validated in animal models and, in a few cases, in clinical trials, further in vivo studies are needed to fully assess the clinical relevance of disease mechanisms and treatments developed for AMD.

### Diabetic retinopathy

Diabetic retinopathy is a result of hyperglycaemia in diabetic patients that leads to atrophy of blood vessels. The initial vascular atrophy restricts blood flow to the retina and causes blood vessel leakage, blurring and creating dark patches in patient’s vision [59]. Treatments for diabetic retinopathy are similar to those for neovascular AMD, as symptoms for both diseases are attributed to retinal vascular defects.

Unlike AMD, loss of cone photoreceptors is not a prominent factor that has been identified in diabetic retinopathy patients [116], and translation of diabetic retinopathy research in cells to clinical presentations may be problematic as cells models lack the aberrant vascularisation that primarily causes the disease. Nonetheless, the effect of hyperglycaemia as well as a range of treatments have been studied in the 661W cone-like cells exposed to high glucose conditions. Proteomic analysis of 661W under hyperglycaemic conditions identified an increase in apoptosis and ROS [117], with another study revealing that 661W cells rely on autophagy to offset these effects [118]. Somatostatin was shown to moderately promote cell survival in diabetic-like 661W conditions [119], whilst sulforaphane has been shown to delay cell death [120], but neither treatment achieved substantial protection of 661W cells. However, in the case of 661W studies, photoreceptor death is induced by high glucose to replicate diabetic conditions, whereas photoreceptor death in the diabetic retina is induced by more complex mechanisms. Another approach taken to mimic diabetic retinopathy conditions is the addition of advanced glycation end products (AGEs), specifically glycated bovine serum albumin (BSA) to 661W cells [121]. AGEs are a diverse family of compounds with a multifaceted role in diabetic retinopathy. Arguably, whether addition of glycated BSA to 661W is representative of the effect of AGEs in diabetic retinopathy, or whether even the addition of AGEs themselves to fully represent the complexity of diabetic retinopathy, should be brought into question. The pathophysiological mechanisms of diabetic retinopathy may therefore be too difficult to fully recapitulate in 661W cells.

### Glaucoma

Glaucoma is a multifactorial optic neuropathy caused by damage to the optic nerve, ultimately resulting in progressive vision loss [59]. Around 80 million people globally are affected by the disease [122]. However, the use of retinal cell lines to study glaucoma has come into question. Previous in vitro work predominantly used the RGC-5 to study glaucoma, as it was the only existing RGC line and was regarded as a breakthrough for glaucoma research at the time [123]. The origin of the RGC-5 was postulated to be developed from 661W cells due to their similarities, though this was later argued to not be the case by different studies [24, 124]. Whether 661W cells could be used as an RGC line was then hypothesised. With the addition of staurosporine, 661W cells were reported to differentiate into retinal ganglion precursor-like cells [125–127]. Although the use of this differentiated 661W line to represent RGCs remains contentious, there is currently no other proliferative cell-based model for optic neuropathies such as glaucoma.

Glaucoma is typically considered an acquired disease, though both genetic and environmental factors contribute to its prevalence. Acquired glaucoma can be simulated in 661W by elevating pressure using a pressure chamber causing pressure-induced toxicity. The therapeutic potential of cannabinoids to lower intraocular pressure has been explored since the 1970s [128]. In a recent study, pressure-induced differentiated 661W cells were able to be rescued with cannabinol, a type of cannabinoid, and further testing in rat models of glaucoma verified cannabinol’s therapeutic effect [126]. Cannabinoids have also shown to prevent degeneration in light-induced stressed 661W cells [129, 130]. While cannabinol has not been evaluated in human glaucoma patients in respect to intraocular pressure, other cannabinoids such as delta-9-tetrahydrocannabinol (THC) and cannabidiol (CBD) show a decrease in intraocular pressure in clinical studies, but therapeutic benefits only lasted a few hours [131–133]. As there are over 100 natural cannabinoids identified and a wide range of synthetic cannabinoids, 661W cells could serve as a valuable tool for screening potential cannabinoid-based glaucoma treatments.

The genetic component of glaucoma has also been explored in 661W cells, specifically the *MYOC* and *OPTN* genes, which are associated with early-onset glaucoma, though both genes are not endogenously expressed in the cell line. Induction of *MYOC* wildtype and mutant gene expression in 661W cells found decrease in autophagic activity, and increased mitochondrial dysfunction and oxidative stress in mutant *MYOC* cells [134]. However, the translatability of this study is uncertain as 661W cells were not differentiated into retinal ganglion precursor-like cells for investigation. In another study, expression of *OPTN* wildtype and mutant gene were induced in differentiated 661W cells, and found to decreased cell viability in cells expressing *OPTN* mutations [125]. Chen et al. (2019) then demonstrated that acteoside, a neuroprotective drug, rescued 661W cells from *OPTN* overexpression-induced death [127]. Acteoside has more recently been investigated in glaucoma-induced animal models and demonstrates promising potential for glaucoma patients [135, 136], but whether acteoside is also beneficial for chronic inherited glaucoma requires further research.

## 661W cells for AAV screening

Adeno-associated viruses (AAV) are commonly used for gene therapy in vision research due to their desirable safety profile, ability to efficiently transduce retinal cells, and their capacity to drive long-term gene expression [137]. The 661W cell line has become a valuable model for testing AAVs, with several studies demonstrating its use in evaluating AAV serotype tropism and transduction efficiency.

Ryals et al. (2011) screened multiple self-complimentary AAVs (scAAVs) for optimal transduction efficiency in 661W and ARPE-19 cells [138]. They identified scAAV1 and scAAV2 to be the most efficient over scAAV5 and scAAV8 vectors and demonstrated that increasing the number of Y-F capsid mutations (the structure that encapsulates the AAV genome) increased transduction efficiency. In 661W cells, a sextuple mutant scAAV2 showed a nine-fold increase in transduction efficiency to unmodified scAAV2 [138]. Another study screened various capsid-mutated AAV vectors in 661W cells and found scAAV2 (quadY-F + T-V) to be the most efficient [139]. These results were further replicated in mice via intravitreal injection, a less targeted but simpler and safer procedure compared to subretinal injections. Remarkably, the addition of quadY-F + T-V mutations to the capsid surface led to ~ 13-fold increase in the number of transduced photoreceptors compared to unmodified scAAV2 [139].

Furthermore, the inner limiting membrane (ILM) can act as a physical barrier, hindering the delivery of AAVs to the photoreceptors via intravitreal injections [140]. Efforts to enhance AAV transduction efficiency were explored by Boye et al. (2016) that developed a sub-inner limiting membrane (subILM) injection technique that significantly improved transgene expression in primate models [141]. To refine this technique, a Healon^®^ injection (a sodium hyaluronate-based ophthalmic viscoelastic) was required to create a chamber for drug administration in the subILM. Healon^®^ with AAV2-smCBA-mCherry was first tested in 661W cells to assess its effects on vector transduction. Preincubation of the AAV2-smCBA-mCherry vector with Healon for varying durations, showed up to a three-fold increase in transduction efficiency after one hour. Importantly, using 661W cells helped validate the vector’s performance before conducting further testing in primate models [141].

An important limitation to consider is the varying tropism of different AAV serotypes for specific cell types, making efficient targeting of the photoreceptors without off-target transduction quite challenging. Previous clinical trials treating retinal dystrophies commonly used AAV2, which could in part be due to the success of the Luxturna^®^ gene therapy to treat RPE cells [3, 142]. However, more research is emerging that indicates AAV2 to preferentially transduce RPE cells over photoreceptors [143, 144]. For example, in subretinally injected non-human primates, AAV2 and AAV8 both efficiently transduced RPE cells at low concentrations, but at higher concentrations were able to target photoreceptors, although AAV8 was significantly better at doing so [143]. Moreover, although testing in 661W cells may offer a rapid screening method, a caveat to consider is that AAV transduction efficiency in vitro cannot directly translate to their performance *in vivo.* AAV5 and AAV8 shown to outperform AAV2 at transducing photoreceptors in vivo despite the popular use of AAV2 [139, 145–148]. In contrast, AAV2 has shown to have superior transduction efficiency in vitro compared to AAV5 and AAV8 [138, 149, 150]. It is made further difficult to address transduction efficiency of AAVs in 661W cells when mean fluorescence intensity of cells is commonly reported in studies, rather than the percentage of positively transduced cells, possibly due to the low transduction of 661W cells [138, 141]. In addition to the ambiguity of transduction efficiency reporting in 661W cells, the intricate retinal architecture and challenges of delivering AAVs to the retina cannot be replicated in 661W cells. Despite these challenges, 661W cells could still be used as preliminary indicator for evaluating AAVs, though these preliminary findings should be paired with further in vivo testing.

Research has also been conducted to integrate the complex and relatively new technology of the CRISPR-Cas9 gene editing into AAVs. Testing CRISPR-Cas9 plasmid transfection in 661W cells fast-tracked the feasibility testing of this system before in vivo applications, which saw restored vision in rhodopsin-deficient mice [151]. As gene editing research is still in its infancy, 661W cells are an ideal model to validate this gene therapy tool along with general AAV screening for feasibility and efficiency in retinal therapies.

## Limitations of 661W cells as a tool to study retinal disease

While 661W cells are a valuable model for studying retinal biology and disease, there are several limitations associated with their use that have been touched on briefly throughout this review. Firstly, the immortalized nature of 661W cells, necessary for their indefinite culture and use in research, can result in deviations from the normal cellular phenotype, including changes in metabolic activity and differentiation states [5]. The 661W cell line undergoes repeated mitosis whilst primary photoreceptors cells are terminally differentiated, with a limited capacity for cell division [152].

661W cells are non-polar unlike photoreceptor cells and, as mentioned earlier, do not form outer segments as they do not express outer segment structural proteins [5]. These alterations may affect the accuracy of reflecting the physiological and pathological characteristics of photoreceptor cells, particularly of those relating to light-mediated processes. Although some studies have shown 661W cells incur physiological changes when exposed to light, the changes are not reflecting of full photoreceptor functionality [8]. Various differentiation elements and manipulations to culture conditions can facilitate inducing immortalized cells to display a more neuronal phenotype, as exemplified in differentiated SH-SY5Y cells that exhibit a more accurate morphological and biological depiction of neurons than when undifferentiated [153, 154]. However, photoreceptors are a highly specialized neuron that may never be able to be fully represented with an immortalized 2-D cell line, though efforts towards bioengineering and cellular reprogramming should be considered to shift the phenotype of 661W cells closer towards a photoreceptor phenotype.

Despite 661W cells being more representative of cone photoreceptors, there is a vast amount of research that use 661W cells as synonymous with all photoreceptors. This is a particular issue when these cells are used to model diseases with mutations in rod-specific genes, as the pathophysiological mechanisms of degeneration may be misrepresented in the cone-like 661W cells. The developments by Huang and colleagues [54] that engineered the 661W-A11 line to be more rod-like has paved the way for future research in rod photoreceptors and their degeneration.

Additionally, while 661W cells express several photoreceptor markers, they do not express all the genes or proteins found in mature photoreceptors, particularly those involved in specific retinal diseases, such as *CNGA3* and *RPE65* [47–49, 81], as discussed earlier. This incomplete representation can limit the utility of 661W cells in fully understanding disease mechanisms and testing therapeutic interventions. Overcoming this limitation by exogenous expression of these genes in 661W cells, particularly of patient-specific mutations, has aided in understanding changes specific mutations have on protein function beyond in silico predictions as well as indicate towards the potential downstream disease mechanisms.

As there is an absence of natural tissue architecture and a lack of practical feedback from other retinal cells, the 661W cell model cannot incorporate all the complexities of retinal disease pathophysiology. This is particularly relevant in the study of acquired retinal diseases such as AMD and diabetic retinopathy. The absence of supporting cells in 661W cell culture, such as Müller glia and retinal pigment epithelium, means that essential aspects of retinal physiology, such as nutrient transport, outer segment renewal, and immune response, are not sufficiently represented. Therefore, results may not correlate with in vivo application accurately, which could lead to disparity between in vitro and in vivo findings. It may be suggested that use of 661W cells in research can only act as preliminary data that later require validation in more complex models, such as animal models. The advantage of initially using 661W cells are that rapid screening of drugs can take place, as well as optimising a dose-response curve that would otherwise require a large number of animals.

Despite these challenges, 661W cells remain a useful model, but their limitations must be considered when interpreting research findings and translating them to clinical contexts. By complementing research that uses immortalized cell lines with further validation, such as in vivo models, researchers can accelerate experimental findings of drug screening and gene therapies without compromising validity of results. Future advancements in bioengineering and cellular reprogramming may further improve the physiological relevance of this widely used model, creating an even more powerful tool for studying photoreceptor degeneration and developing novel therapeutics.

## Conclusion

The 661W cell line has emerged as an invaluable tool for investigating both inherited and acquired retinal diseases, significantly advancing our understanding of the pathogenesis, molecular mechanisms, and potential therapeutic strategies for these conditions. This cell line offers the ability to control cell conditions, is a more affordable system when compared to animal research, and has stable culturing conditions, allowing for consistency across experiments. Together, these factors offer the ability to assess cone cell death, differentiation, cytotoxicity, drug efficacy, and much more. Studies utilising the 661W cell line have been instrumental in elucidating the underlying mechanisms of single mutation inherited retinal diseases, paving the way for treatment development. However, the multifaceted and complex nature of acquired retinal diseases poses challenges in accurately recapitulating these conditions in the 661W model. It is crucial to acknowledge these limitations and recognize the importance of translating findings from the cell line to in vivo models and human patients. Moving forward, future studies should prioritize integrating findings from the 661W cell line with in vivo models to validate the translational relevance of these studies. This approach will not only enhance our understanding of disease mechanisms, but also accelerate the development of effective treatments for retinal diseases. The 661W cell line has facilitated our understanding of retinal diseases, and holds promise for further advancements in unravelling their disease mechanisms and developing innovative therapeutic interventions.

## Data Availability

Not applicable.

## References

[1] Crewe JM, Morlet N, Morgan WH, Spilsbury K, Mukhtar AS, Clark A, Semmens JB. Mortality and hospital morbidity of working-age blind. Br J Ophthalmol. 2013;97(12):1579–85. 24123905 10.1136/bjophthalmol-2013-303993

[2] Prem Senthil M, Khadka J, Gilhotra JS, Simon S, Pesudovs K. Exploring the quality of life issues in people with retinal diseases: a qualitative study. J patient-reported Outcomes. 2017;1:1–14. 10.1186/s41687-017-0023-4 PMC5934910 29757297

[3] Food US, Administation D. LUXTURNA 2017 [Available from: https://www.fda.gov/vaccines-blood-biologics/cellular-gene-therapy-products/luxturna

[4] Al-Ubaidi MR, Font RL, Quiambao AB, Keener MJ, Liou GI, Overbeek PA, Baehr W. Bilateral retinal and brain tumors in Transgenic mice expressing Simian virus 40 large T antigen under control of the human interphotoreceptor retinoid-binding protein promoter. J Cell Biol. 1992;119(6):1681–7. 1334963 10.1083/jcb.119.6.1681 PMC2289740

[5] Tan E, Ding X-Q, Saadi A, Agarwal N, Naash MI, Al-Ubaidi MR. Expression of cone-photoreceptor–specific antigens in a cell line derived from retinal tumors in Transgenic mice. Investig Ophthalmol Vis Sci. 2004;45(3):764–8. 14985288 10.1167/iovs.03-1114 PMC2937568

[6] Al-Ubaidi MR, Hollyfield JG, Overbeek PA, Baehr W. Photoreceptor degeneration induced by the expression of Simian virus 40 large tumor antigen in the retina of Transgenic mice. Proc Natl Acad Sci. 1992;89(4):1194–8. 10.1073/pnas.89.4.1194 PMC48415 1311085

[7] Moon SY, Zhang D, Chen S-C, Lamey TM, Thompson JA, McLaren TL, De Roach JN, Chen FK, McLenachan S. Generation of two induced pluripotent stem cell lines from a retinitis pigmentosa patient with compound heterozygous mutations in CRB1. Stem Cell Res. 2021;54:102403. 34034222 10.1016/j.scr.2021.102403

[8] Zhang Y, Yin R, Liu X. Changes in Cyclic Guanosine monophosphate channel of 661w cells in vitro with excessive light time. J Ophthalmic Vis Res. 2023;18(4):417. 38250228 10.18502/jovr.v18i4.14554 PMC10794799

[9] Chen W-J, Wu C, Xu Z, Kuse Y, Hara H, Duh EJ. Nrf2 protects photoreceptor cells from photo-oxidative stress induced by blue light. Exp Eye Res. 2017;154:151–8. 27923559 10.1016/j.exer.2016.12.001 PMC6054877

[10] Lin C-W, Yang C-M, Yang C-H. Protective effect of Astaxanthin on blue light light-emitting diode-induced retinal cell damage via free radical scavenging and activation of PI3K/Akt/Nrf2 pathway in 661W cell model. Mar Drugs. 2020;18(8):387. 32722441 10.3390/md18080387 PMC7459684

[11] Natoli R, Rutar M, Lu Y-Z, Chu-Tan JA, Chen Y, Saxena K, Madigan M, Valter K, Provis JM. The role of pyruvate in protecting 661W photoreceptor-like cells against light-induced cell death. Curr Eye Res. 2016;41(11):1473–81. 27217092 10.3109/02713683.2016.1139725

[12] Xia H, Hu Q, Li L, Tang X, Zou J, Huang L, Li X. Protective effects of autophagy against blue light-induced retinal degeneration in aged mice. Sci China Life Sci. 2019;62:244–56. 30238280 10.1007/s11427-018-9357-y

[13] Wheway G, Nazlamova L, Turner D, Cross S. 661W photoreceptor cell line as a cell model for studying retinal ciliopathies. Front Genet. 2019;10:308. 31024622 10.3389/fgene.2019.00308 PMC6459963

[14] Reid TW, Albert DM, Rabson AS, Russell P, Craft J, Chu EW, Tralka TS, Wilcox JL. Characteristics of an established cell line of retinoblastoma. J Natl Cancer Inst. 1974;53(2):347–60. 4135597 10.1093/jnci/53.2.347

[15] McFall RC, Sery TW, Makadon M. Characterization of a new continuous cell line derived from a human retinoblastoma. Cancer Res. 1977;37(4):1003–10. 844036

[16] Gómez-Vicente V, Flores A, Lax P, Murciano C, Yáñez A, Gil ML, Cuenca N, Gozalbo D, Maneu V. Characterization of a new murine retinal cell line (MU-PH1) with glial, progenitor and photoreceptor characteristics. Exp Eye Res. 2013;110:125–35. 23375594 10.1016/j.exer.2012.12.006

[17] Di Polo A, Farber DB. Rod photoreceptor-specific gene expression in human retinoblastoma cells. Proceedings of the National Academy of Sciences. 1995;92(9):4016-20. 10.1073/pnas.92.9.4016 PMC42093 7732024

[18] Bazhin AV, Schadendorf D, Owen RW, Zernii EY, Philippov PP, Eichmuller SB. Visible light modulates the expression of cancer-retina antigens. Mol Cancer Res. 2008;6(1):110–8. 18184973 10.1158/1541-7786.MCR-07-0140

[19] Liu Y, Hu H, Liang M, Xiong Y, Li K, Chen M, Fan Z, Kuang X, Deng F, Liu X. Regulated differentiation of WERI-Rb-1 cells into retinal neuron-like cells. Int J Mol Med. 2017;40(4):1172–84. 28848998 10.3892/ijmm.2017.3102 PMC5593461

[20] Deeb S, Bisset D, Fu L. Epigenetic control of expression of the human L-and M‐pigment genes. Ophthalmic Physiol Opt. 2010;30(5):446–53. 20883327 10.1111/j.1475-1313.2010.00735.x

[21] Sparrrow R, Hicks J, Hamel DP. The retinal pigment epithelium in health and disease. Curr Mol Med. 2010;10(9):802–23. 21091424 10.2174/156652410793937813 PMC4120883

[22] Dunn K, Aotaki-Keen A, Putkey F, Hjelmeland L. ARPE-19, a human retinal pigment epithelial cell line with differentiated properties. Exp Eye Res. 1996;62(2):155–70. 8698076 10.1006/exer.1996.0020

[23] Krishnamoorthy R, Agarwal P, Prasanna G, Vopat K, Lambert W, Sheedlo H, Pang I-H, Shade D, Wordinger R, Yorio T. RETRACTED: characterization of a transformed rat retinal ganglion cell line. Elsevier; 2001. 10.1016/s0169-328x(00)00224-2 11165366

[24] Hurst J, Attrodt G, Bartz-Schmidt K-U, Mau-Holzmann UA, Spitzer MS, Schnichels S. A case study from the past:the RGC-5 vs. the 661W cell line: similarities, differences and Contradictions—Are they really the same?? Int J Mol Sci. 2023;24(18):13801. 37762103 10.3390/ijms241813801 PMC10531351

[25] Limb GA, Salt TE, Munro PM, Moss SE, Khaw PT. In vitro characterization of a spontaneously immortalized human Muller cell line (MIO-M1). Investig Ophthalmol Vis Sci. 2002;43(3):864–9. 11867609

[26] Ohsawa K, Imai Y, Nakajima K, Kohsaka S. Generation and characterization of a microglial cell line, MG5, derived from a p53-deficient mouse. Glia. 1997;21(3):285–98. 9383038

[27] Moscona A. Rotation-mediated histogenetic aggregation of dissociated cells: a quantifiable approach to cell interactions in vitro. Exp Cell Res. 1961;22:455–75. 13773012 10.1016/0014-4827(61)90122-7

[28] Laabich A, Vissvesvaran GP, Lieu KL, Murata K, McGinn TE, Manmoto CC, Sinclair JR, Karliga I, Leung DW, Fawzi A, Kubota R. Protective effect of Crocin against blue Light – and white Light–Mediated photoreceptor cell death in bovine and primate retinal primary cell culture. Investig Ophthalmol Vis Sci. 2006;47(7):3156–63. 16799063 10.1167/iovs.05-1621

[29] Maisto R, Gesualdo C, Trotta MC, Grieco P, Testa F, Simonelli F, Barcia JM, D’Amico M, Di Filippo C, Rossi S. Melanocortin receptor agonists MCR 1-5 protect photoreceptors from high‐glucose damage and restore antioxidant enzymes in primary retinal cell culture. J Cell Mol Med. 2017;21(5):968–74. 27998021 10.1111/jcmm.13036 PMC5387132

[30] Leung DW, Lindlief LA, Laabich A, Vissvesvaran GP, Kamat M, Lieu KL, Fawzi A, Kubota R. Minocycline protects photoreceptors from light and oxidative stress in primary bovine retinal cell culture. Investig Ophthalmol Vis Sci. 2007;48(1):412–21. 17197562 10.1167/iovs.06-0522

[31] Skaper SD. Isolation and culture of rat cone photoreceptor cells. In: Skaper SD, editor. Neurotrophic factors: methods protocols. New York: Humana Press; 2012. p. 147–58. 10.1007/978-1-61779-536-7_13 22367808

[32] Forouzanfar F, Shojapour M, Aghili ZS, Asgharzade S. Growth factors as tools in photoreceptor cell regeneration and vision recovery. Curr Drug Targets. 2020;21(6):573–81. 31755378 10.2174/1389450120666191121103831

[33] Zhang X, Wang W, Jin Z-B. Retinal organoids as models for development and diseases. Cell Regeneration. 2021;10:1–10. 34719743 10.1186/s13619-021-00097-1 PMC8557999

[34] McLenachan S, Zhang D, Grainok J, Zhang X, Huang Z, Chen S-C, Zaw K, Lima A, Jennings L, Roshandel D. Determinants of disease penetrance in PRPF31-associated retinopathy. Genes. 2021;12(10):1542. 34680937 10.3390/genes12101542 PMC8535263

[35] Moon SY, Zhang D, Chen S-C, Lamey TM, Thompson JA, McLaren TL, Chen FK, McLenachan S. Rapid variant pathogenicity analysis by CRISPR activation of CRB1 gene expression in patient-derived fibroblasts. CRISPR J. 2024;7(2):100–10. 38579141 10.1089/crispr.2023.0065

[36] Mellough CB, Collin J, Queen R, Hilgen G, Dorgau B, Zerti D, Felemban M, White K, Sernagor E, Lako M. Systematic comparison of retinal organoid differentiation from human pluripotent stem cells reveals stage specific, cell line, and methodological differences. Stem Cells Transl Med. 2019;8(7):694–706. 30916455 10.1002/sctm.18-0267 PMC6591558

[37] Gasparini SJ, Llonch S, Borsch O, Ader M. Transplantation of photoreceptors into the degenerative retina: current state and future perspectives. Prog Retin Eye Res. 2019;69:1–37. 30445193 10.1016/j.preteyeres.2018.11.001

[38] Kohl S, Jägle H, Wissinger B, Zobor D, Achromatopsia. GeneReviews^®^[Internet]. 2018.

[39] Kohl S, Jägle H, Wissinger B, Zobor D. Achromatopsia. In: Adam MP, Ardinger HH, Pagon RA, Wallace SE, Bean LJH, Stephens K, et al. editors. GeneReviews(^®^). Seattle (WA): University of Washington, Seattle; 2018. 20301591

[40] Retinal Information Network [Internet]. 2024. Available from: https://sph.uth.edu/retnet/

[41] Zelinger L, Cideciyan AV, Kohl S, Schwartz SB, Rosenmann A, Eli D, Sumaroka A, Roman AJ, Luo X, Brown C. Genetics and disease expression in the CNGA3 form of achromatopsia: steps on the path to gene therapy. Ophthalmology. 2015;122(5):997–1007. 25616768 10.1016/j.ophtha.2014.11.025

[42] Li S, Huang L, Xiao X, Jia X, Guo X, Zhang Q. Identification of CNGA3 mutations in 46 families: common cause of achromatopsia and cone-rod dystrophies in Chinese patients. JAMA Ophthalmol. 2014;132(9):1076–83. 24903488 10.1001/jamaophthalmol.2014.1032

[43] Reichel FF, Michalakis S, Wilhelm B, Zobor D, Muehlfriedel R, Kohl S, Weisschuh N, Sothilingam V, Kuehlewein L, Kahle N. Three-year results of phase I retinal gene therapy trial for CNGA3-mutated achromatopsia: results of a Non randomised controlled trial. Br J Ophthalmol. 2022;106(11):1567–72. 34006508 10.1136/bjophthalmol-2021-319067

[44] Michaelides M, Hirji N, Wong SC, Besirli CG, Zaman S, Kumaran N, Georgiadis A, Smith AJ, Ripamonti C, Gottlob I. First-in-human gene therapy trial of AAV8-hCARp. hCNGB3 in adults and children with CNGB3-associated achromatopsia. Am J Ophthalmol. 2023;253:243–51. 37172884 10.1016/j.ajo.2023.05.009

[45] Ye G-J, Budzynski E, Sonnentag P, Nork TM, Sheibani N, Gurel Z, Boye SL, Peterson JJ, Boye SE, Hauswirth WW. Cone-specific promoters for gene therapy of achromatopsia and other retinal diseases. Hum Gene Ther. 2016;27(1):72–82. 26603570 10.1089/hum.2015.130 PMC4741229

[46] Fitzgerald JB, Malykhina AP, Al-Ubaidi MR, Ding X-Q. Functional expression of cone cyclic nucleotide-gated channel in cone photoreceptor-derived 661W cells. In: Anderson RE, LaVail MM, Hollyfield JG, editors. Recent advances in retinal degeneration. New York: Springer; 2008. p. 327–34. 10.1007/978-0-387-74904-4_38 18188961

[47] Zheng X, Li H, Hu Z, Su D, Yang J. Structural and functional characterization of an achromatopsia-associated mutation in a phototransduction channel. Commun Biology. 2022;5(1):190. 10.1038/s42003-022-03120-6 PMC8888761 35233102

[48] Liu C, Sherpa T, Varnum MD. Disease-associated mutations in CNGB3 promote cytotoxicity in photoreceptor-derived cells. Mol Vis. 2013;19:1268. 23805033 10.1167/13.9.1268 PMC3692405

[49] Duricka DL, Brown RL, Varnum MD. Defective trafficking of cone photoreceptor CNG channels induces the unfolded protein response and ER-stress-associated cell death. Biochem J. 2012;441(2):685–96. 21992067 10.1042/BJ20111004 PMC3257030

[50] Michalakis S, Geiger H, Haverkamp S, Hofmann F, Gerstner A, Biel M. Impaired Opsin targeting and cone photoreceptor migration in the retina of mice lacking the Cyclic nucleotide-gated channel CNGA3. Investig Ophthalmol Vis Sci. 2005;46(4):1516–24. 15790924 10.1167/iovs.04-1503

[51] Arango-Gonzalez B, Trifunović D, Sahaboglu A, Kranz K, Michalakis S, Farinelli P, Koch S, Koch F, Cottet S, Janssen-Bienhold U. Identification of a common non-apoptotic cell death mechanism in hereditary retinal degeneration. PLoS ONE. 2014;9(11):e112142. 25392995 10.1371/journal.pone.0112142 PMC4230983

[52] Sancho-Pelluz J, Arango-Gonzalez B, Kustermann S, Romero FJ, Van Veen T, Zrenner E, Ekström P, Paquet-Durand F. Photoreceptor cell death mechanisms in inherited retinal degeneration. Mol Neurobiol. 2008;38(3):253–69. 18982459 10.1007/s12035-008-8045-9

[53] Choi YJ, Joo K, Lim HT, Kim SS, Han J, Woo SJ. Clinical and genetic features of Korean patients with achromatopsia. Genes. 2023;14(2):519. 36833446 10.3390/genes14020519 PMC9957537

[54] Huang L, Kutluer M, Adani E, Comitato A, Marigo V. New in vitro cellular model for molecular studies of retinitis pigmentosa. Int J Mol Sci. 2021;22(12):6440. 34208617 10.3390/ijms22126440 PMC8235468

[55] Wolf A, Aslanidis A, Langmann T. Retinal expression and localization of Mef2c support its important role in photoreceptor gene expression. Biochem Biophys Res Commun. 2017;483(1):346–51. 28017720 10.1016/j.bbrc.2016.12.141

[56] Li G-Y, Fan B, Jiao Y-Y. Rapamycin attenuates visible light-induced injury in retinal photoreceptor cells via inhibiting Endoplasmic reticulum stress. Brain Res. 2014;1563:1–12. 24607296 10.1016/j.brainres.2014.02.020

[57] Wang AL, Knight DK, Thanh-thao TV, Mehta MC. Retinitis pigmentosa: review of current treatment. Int Ophthalmol Clin. 2019;59(1):263–80. 30585930 10.1097/IIO.0000000000000256

[58] Meunier I, Arndt C, Zanlonghi X, Defoort-Dhellemmes S, Drumare I, Mauget-Faÿsse M, Wolff B, Affortit A, Hamel C, Puech B. Spectral-Domain Optical Coherence Tomography in Hereditary Retinal Dystrophies. 2012.

[59] Pardue MT, Allen RS. Neuroprotective strategies for retinal disease. Prog Retin Eye Res. 2018;65:50–76. 29481975 10.1016/j.preteyeres.2018.02.002 PMC6081194

[60] Brunet AA, Harvey AR, Carvalho LS. Primary and secondary cone cell death mechanisms in inherited retinal diseases and potential treatment options. Int J Mol Sci. 2022;23(2):726. 35054919 10.3390/ijms23020726 PMC8775779

[61] Fabiani C, Zulueta A, Bonezzi F, Casas J, Ghidoni R, Signorelli P, Caretti A. 2-Acetyl-5-tetrahydroxybutyl imidazole (THI) protects 661W cells against oxidative stress. Naunyn Schmiedebergs Arch Pharmacol. 2017;390:741–51. 28409209 10.1007/s00210-017-1374-3

[62] Tahia F, Basu SK, Prislovsky A, Mondal K, Ma D, Kochat H, Brown K, Stephenson DJ, Chalfant CE, Mandal N. Sphingolipid biosynthetic inhibitor L-Cycloserine prevents oxidative-stress-mediated death in an in vitro model of photoreceptor-derived 661W cells. Exp Eye Res. 2024;242:109852. 38460719 10.1016/j.exer.2024.109852 PMC11089890

[63] Zhu P, Yang Q, Li G, Chang Q. PKM2 Is a Potential Diagnostic and Therapeutic Target for Retinitis Pigmentosa. Disease Markers. 2021;2021. 10.1155/2021/1602797 PMC8601838 34804260

[64] Leyk J, Daly C, Janssen-Bienhold U, Kennedy BN, Richter-Landsberg C. HDAC6 Inhibition by Tubastatin A is protective against oxidative stress in a photoreceptor cell line and restores visual function in a zebrafish model of inherited blindness. Cell Death Dis. 2017;8(8):e3028–e. 29048427 10.1038/cddis.2017.415 PMC5596594

[65] Kunchithapautham K, Rohrer B. Apoptosis and autophagy in photoreceptors exposed to oxidative stress. Autophagy. 2007;3(5):433–41. 17471016 10.4161/auto.4294

[66] Kihara A, Mitsutake S, Mizutani Y, Igarashi Y. Metabolism and biological functions of two phosphorylated sphingolipids, sphingosine 1-phosphate and ceramide 1-phosphate. Prog Lipid Res. 2007;46(2):126–44. 17449104 10.1016/j.plipres.2007.03.001

[67] Miller AL, James RE, Harvey AR, Trifunović D, Carvalho LS. The role of epigenetic changes in the pathology and treatment of inherited retinal diseases. Front Cell Dev Biology. 2023;11:1224078. 10.3389/fcell.2023.1224078 PMC10436478 37601102

[68] Perron NR, Nasarre C, Bandyopadhyay M, Beeson CC, Rohrer B. SAHA is neuroprotective in in vitro and in situ models of retinitis pigmentosa. Mol Vis. 2021;27:151–60. PMC8056468 33907370

[69] Arroba AI, Wallace D, Mackey A, De La Rosa EJ, Cotter TG. IGF-I maintains Calpastatin expression and attenuates apoptosis in several models of photoreceptor cell death. Eur J Neurosci. 2009;30(6):975–86. 19723289 10.1111/j.1460-9568.2009.06902.x

[70] Luodan A, Zou T, He J, Chen X, Sun D, Fan X, Xu H. Rescue of retinal degeneration in rd1 mice by intravitreally injected Metformin. Front Mol Neurosci. 2019;12. 10.3389/fnmol.2019.00102 PMC6497809 31080404

[71] Punzo C, Kornacker K, Cepko CL. Stimulation of the Insulin/mTOR pathway delays cone death in a mouse model of retinitis pigmentosa. Nat Neurosci. 2009;12(1):44. 19060896 10.1038/nn.2234 PMC3339764

[72] Venkatesh A, Ma S, Le YZ, Hall MN, Rüegg MA, Punzo C. Activated mTORC1 promotes long-term cone survival in retinitis pigmentosa mice. J Clin Investig. 2015;125(4):1446–58. 25798619 10.1172/JCI79766 PMC4396488

[73] Lin B, Xiong G, Yang W. Ribosomal protein S6 kinase 1 promotes the survival of photoreceptors in retinitis pigmentosa. Cell Death Dis. 2018;9(12):1141. 30442943 10.1038/s41419-018-1198-1 PMC6237824

[74] Liu Y, Wang X, Gong R, Xu G, Zhu M. Overexpression of rhodopsin or its mutants leads to energy metabolism dysfunction in 661w cells. Invest Ophthalmol Vis Sci. 2022;63(13):2. 36469028 10.1167/iovs.63.13.2 PMC9730732

[75] Oh EC, Cheng H, Hao H, Jia L, Khan NW, Swaroop A. Rod differentiation factor NRL activates the expression of nuclear receptor NR2E3 to suppress the development of cone photoreceptors. Brain Res. 2008;1236:16–29. 18294621 10.1016/j.brainres.2008.01.028 PMC2660138

[76] Sahaboglu A, Paquet-Durand O, Dietter J, Dengler K, Bernhard-Kurz S, Ekström PA, Hitzmann B, Ueffing M, Paquet-Durand F. Retinitis pigmentosa: rapid neurodegeneration is governed by slow cell death mechanisms. Cell Death Dis. 2013;4(2):e488–e. 23392176 10.1038/cddis.2013.12 PMC3593146

[77] Mencl S, Trifunović D, Zrenner E, Paquet-Durand F, editors. PKG-dependent cell death in 661W cone photoreceptor-like cell cultures (experimental study). Retinal Degenerative Diseases: Mechanisms and Experimental Therapy;; 2018. 10.1007/978-3-319-75402-4_63 29721983

[78] Den Hollander AI, Roepman R, Koenekoop RK, Cremers FP. Leber congenital amaurosis: genes, proteins and disease mechanisms. Prog Retin Eye Res. 2008;27(4):391–419. 18632300 10.1016/j.preteyeres.2008.05.003

[79] Sallum JM, Kaur VP, Shaikh J, Banhazi J, Spera C, Aouadj C, Viriato D, Fischer MD. Epidemiology of mutations in the 65-kDa retinal pigment epithelium (RPE65) gene-mediated inherited retinal dystrophies: a systematic literature review. Adv Therapy. 2022;39(3):1179–98. 10.1007/s12325-021-02036-7 PMC8918161 35098484

[80] Tang PH, Buhusi MC, Ma J-X, Crouch RK. RPE65 is present in human green/red cones and promotes photopigment regeneration in an in vitro cone cell model. J Neurosci. 2011;31(50):18618–26. 22171060 10.1523/JNEUROSCI.4265-11.2011 PMC3297673

[81] Minegishi Y, Sheng X, Yoshitake K, Sergeev Y, Iejima D, Shibagaki Y, Monma N, Ikeo K, Furuno M, Zhuang W. CCT2 mutations evoke leber congenital amaurosis due to chaperone complex instability. Sci Rep. 2016;6(1):33742. 27645772 10.1038/srep33742 PMC5028737

[82] Castiglione A, Möller C. Usher syndrome. Audiol Res. 2022;12(1):42–65. 35076463 10.3390/audiolres12010005 PMC8788290

[83] French LS, Mellough CB, Chen FK, Carvalho LS. A review of gene, drug and cell-based therapies for Usher syndrome. Front Cell Neurosci. 2020;14:183. 32733204 10.3389/fncel.2020.00183 PMC7363968

[84] Géléoc GG, El-Amraoui A. Disease mechanisms and gene therapy for Usher syndrome. Hear Res. 2020;394:107932. 32199721 10.1016/j.heares.2020.107932

[85] Ryu J, Statz JP, Chan W, Burch FC, Brigande JV, Kempton B, Porsov EV, Renner L, McGill T, Burwitz BJ. CRISPR/Cas9 editing of the MYO7A gene in rhesus macaque embryos to generate a primate model of Usher syndrome type 1B. Sci Rep. 2022;12(1):10036. 35710827 10.1038/s41598-022-13689-x PMC9203743

[86] Panagiotopoulos A-L, Karguth N, Pavlou M, Böhm S, Gasparoni G, Walter J, Graf A, Blum H, Biel M, Riedmayr LM. Antisense oligonucleotide-and CRISPR-Cas9-mediated rescue of mRNA splicing for a deep intronic CLRN1 mutation. Mol Therapy-Nucleic Acids. 2020;21:1050–61. 10.1016/j.omtn.2020.07.036 PMC7452116 32841912

[87] Forsythe E, Beales PL. Bardet–biedl syndrome. Eur J Hum Genet. 2013;21(1):8–13. 22713813 10.1038/ejhg.2012.115 PMC3522196

[88] Berezovsky A, Rocha DM, Sacai PY, Watanabe SS, Cavascan NN, Salomao SR. Visual acuity and retinal function in patients with Bardet-Biedl syndrome. Clinics. 2012;67(2):145–9. 22358239 10.6061/clinics/2012(02)09 PMC3275121

[89] Weihbrecht K, Goar WA, Pak T, Garrison JE, DeLuca AP, Stone EM, Scheetz TE, Sheffield VC. Keeping an eye on Bardet-Biedl syndrome: a comprehensive review of the role of Bardet-Biedl syndrome genes in the eye. Med Res Archives. 2017;5(9). 10.18103/mra.v5i9.1526 PMC5814251 29457131

[90] National Organization for Rare Disorders. Bardet-Biedl Syndrome 2022 [Available from: https://rarediseases.org/rare-diseases/bardet-biedl-syndrome/#causes

[91] Zhang C-J, Xiang L, Chen X-J, Wang X-Y, Wu K-C, Zhang B-W, Chen D-F, Jin G-H, Zhang H, Chen Y-C. Ablation of mature miR-183 leads to retinal dysfunction in mice. Investig Ophthalmol Vis Sci. 2020;61(3):12. 10.1167/iovs.61.3.12 PMC7401733 32176259

[92] Lai T-T, Yang C-M, Yang C-H. Astaxanthin protects retinal photoreceptor cells against high glucose-induced oxidative stress by induction of antioxidant enzymes via the PI3K/Akt/Nrf2 pathway. Antioxidants. 2020;9(8):729. 32785112 10.3390/antiox9080729 PMC7465141

[93] He D, Tao L, Cai B, Chen X, Wang Y, Li S, Liao C, Chen Y, Chen J, Liu Z. eIF2α incites photoreceptor cell and retina damage by all-trans-retinal. J Biol Chem. 2023;299(5). 10.1016/j.jbc.2023.104686 PMC10193240 37031820

[94] Yang B, Yang K, Chen J, Wu Y. Crocin protects the 661W murine photoreceptor cell line against the toxic effects of All-Trans-Retinal. Int J Mol Sci. 2024;25(18):10124. 39337609 10.3390/ijms251810124 PMC11432120

[95] Liou J-C, Yang S-L, Wang P-H, Wu J-L, Huang Y-P, Chen B-Y, Lee M-C. Protective effect of Crocin against the declining of high Spatial frequency-based visual performance in mice. J Funct Foods. 2018;49:314–23.

[96] Du J, Huang Y, Li K, Yu X, Jin H, Yang L. Retina-derived endogenous sulfur dioxide might be a novel anti-apoptotic factor. Biochem Biophys Res Commun. 2018;496(3):955–60. 10.1016/j.bbrc.2018.01.103 29402407

[97] Dong S-Q, Xu H-Z, Xia X-B, Wang S, Zhang L-X, Liu S-Z. Activation of the ERK 1/2 and STAT3 signaling pathways is required for 661W cell survival following oxidant injury. Int J Ophthalmol. 2012;5(2):138. 22762037 10.3980/j.issn.2222-3959.2012.02.04 PMC3359025

[98] Ma W, Zhu X, Ding X, Li T, Hu Y, Hu X, Yuan L, Lei L, Hu A, Luo Y. Protective effects of SS31 on t–BHP induced oxidative damage in 661W cells. Mol Med Rep. 2015;12(4):5026–34. 26165373 10.3892/mmr.2015.4055 PMC4581771

[99] Mandal MNA, Patlolla JM, Zheng L, Agbaga M-P, Tran J-TA, Wicker L, Kasus-Jacobi A, Elliott MH, Rao CV, Anderson RE. Curcumin protects retinal cells from light-and oxidant stress-induced cell death. Free Radic Biol Med. 2009;46(5):672–9. 19121385 10.1016/j.freeradbiomed.2008.12.006 PMC2810836

[100] Piermarocchi S, Saviano S, Parisi V, Tedeschi M, Panozzo G, Scarpa G, Boschi G, Lo Giudice G. Carotenoids in Age-related maculopathy Italian study (CARMIS): two-year results of a randomized study. Eur J Ophthalmol. 2012;22(2):216. 22009916 10.5301/ejo.5000069

[101] Parisi V, Tedeschi M, Gallinaro G, Varano M, Saviano S, Piermarocchi S, Group CS. Carotenoids and antioxidants in age-related maculopathy Italian study: multifocal electroretinogram modifications after 1 year. Ophthalmology. 2008;115(2):324–33. e2. 17716735 10.1016/j.ophtha.2007.05.029

[102] Rosen R, Hu D-N, Perez V, Tai K, Yu G-P, Chen M, Tone P, McCormick SA, Walsh J. Urinary 6-sulfatoxymelatonin level in age-related macular degeneration patients. Mol Vis. 2009;15:1673. 19710945 PMC2730752

[103] Yi C, Pan X, Yan H, Guo M, Pierpaoli W. Effects of melatonin in age-related macular degeneration. Ann N Y Acad Sci. 2005;1057(1):384–92. 16399908 10.1196/annals.1356.029

[104] Sánchez-Bretaño A, Baba K, Janjua U, Piano I, Gargini C, Tosini G. Melatonin partially protects 661W cells from H2O2-induced death by inhibiting Fas/FasL-caspase-3. Mol Vis. 2017;23:844. 29259391 PMC5723148

[105] Baba K, Suen T-C, Goyal V, Stowie A, Davidson A, DeBruyne J, Tosini G. The circadian clock mediates the response to oxidative stress in a cone photoreceptor–like (661W) cell line via regulation of glutathione peroxidase activity. F1000Research. 2022;11:1072. 10.12688/f1000research.125133.1 PMC9639596 36405557

[106] Shi H, Williams JA, Guo L, Stampoulis D, Francesca Cordeiro M, Moss SE. Exposure to the complement C5b-9 complex sensitizes 661W photoreceptor cells to both apoptosis and necroptosis. Apoptosis. 2015;20:433–43. 25735751 10.1007/s10495-015-1091-7 PMC4348505

[107] Lee M, Yun S, Lee H, Yang J. Quercetin mitigates inflammatory responses induced by vascular endothelial growth factor in mouse retinal photoreceptor cells through suppression of nuclear factor kappa B. Int J Mol Sci. 2017;18(11):2497. 29165402 10.3390/ijms18112497 PMC5713462

[108] Schnichels S, Hagemann U, Januschowski K, Hofmann J, Bartz-Schmidt K-U, Szurman P, Spitzer MS, Aisenbrey S. Comparative toxicity and proliferation testing of Aflibercept, bevacizumab and Ranibizumab on different ocular cells. Br J Ophthalmol. 2013;97(7):917–23. 23686000 10.1136/bjophthalmol-2013-303130

[109] Ortega JT, Parmar T, Golczak M, Jastrzebska B. Protective effects of flavonoids in acute models of light-induced retinal degeneration. Mol Pharmacol. 2021;99(1):60–77. 33154094 10.1124/molpharm.120.000072 PMC7736834

[110] Kamoshita M, Fujinami K, Toda E, Tsubota K, Ozawa Y. Neuroprotective effect of activated 5′-adenosine monophosphate-activated protein kinase on cone system function during retinal inflammation. BMC Neurosci. 2016;17:1–6. 27287531 10.1186/s12868-016-0268-5 PMC4902963

[111] Sun Y, Lin Z, Liu C-H, Gong Y, Liegl R, Fredrick TW, Meng SS, Burnim SB, Wang Z, Akula JD. Inflammatory signals from photoreceptor modulate pathological retinal angiogenesis via c-Fos. J Exp Med. 2017;214(6):1753–67. 28465464 10.1084/jem.20161645 PMC5461000

[112] Parmeggiani F, Romano MR, Costagliola C, Semeraro F, Incorvaia C, D’Angelo S, Perri P, De Palma P, De Nadai K, Sebastiani A. Mechanism of inflammation in age-related macular degeneration. Mediat Inflamm. 2012;2012(1):546786. 10.1155/2012/546786 PMC3504473 23209345

[113] Kawa MP, Machalinska A, Roginska D, Machalinski B. Complement system in pathogenesis of AMD: dual player in degeneration and protection of retinal tissue. J Immunol Res. 2014;2014(1):483960. 25276841 10.1155/2014/483960 PMC4168147

[114] Wong RW, D CHIMENE R, Hahn P, Green WR, Dunaief JL. Iron toxicity as a potential factor in AMD. Retina. 2007;27(8):997–1003. 18040235 10.1097/IAE.0b013e318074c290

[115] Huang H, Zeng J, Yu X, Du H, Wen C, Mao Y, Tang H, Kuang X, Liu W, Yu H. Establishing chronic models of age-related macular degeneration via long-term iron ion overload. Am J Physiology-Cell Physiol. 2024;326(5):C1367–83. 10.1152/ajpcell.00532.2023 38406826

[116] Tonade D, Kern TS. Photoreceptor cells and RPE contribute to the development of diabetic retinopathy. Prog Retin Eye Res. 2021;83:100919. 33188897 10.1016/j.preteyeres.2020.100919 PMC8113320

[117] Lam CH-I, Cheung JK-W, Tse DY-Y, Lam TC. Proteomic profiling revealed mitochondrial dysfunction in photoreceptor cells under hyperglycemia. Int J Mol Sci. 2022;23(21):13366. 36362154 10.3390/ijms232113366 PMC9658613

[118] Taki K, Horie T, Kida T, Mimura M, Ikeda T, Oku H. Impairment of autophagy causes superoxide formation and caspase activation in 661W cells, a cell line for cone photoreceptors, under hyperglycemic conditions. Int J Mol Sci. 2020;21(12):4240. 32545902 10.3390/ijms21124240 PMC7352513

[119] Arroba AI, Mazzeo A, Cazzoni D, Beltramo E, Hernández C, Porta M, Simó R, Valverde ÁM. Somatostatin protects photoreceptor cells against high glucose–induced apoptosis. Mol Vis. 2016;22:1522. 28050125 PMC5204461

[120] Lv J, Bao S, Liu T, Wei L, Wang D, Ye W, Wang N, Song S, Li J, Chudhary M. Sulforaphane delays diabetes-induced retinal photoreceptor cell degeneration. Cell Tissue Res. 2020;382:477–86. 32783101 10.1007/s00441-020-03267-w

[121] Song S, Bao S, Zhang C, Zhang J, Lv J, Li X, Chudhary M, Ren X, Kong L. Stimulation of AMPK prevents diabetes-induced photoreceptor cell degeneration. Oxidative Med Cell Longev. 2021;2021(1):5587340. 10.1155/2021/5587340 PMC8140850 34093959

[122] Allison K, Patel D, Alabi O. Epidemiology of glaucoma: the past, present, and predictions for the future. Cureus. 2020;12(11). 10.7759/cureus.11686 PMC7769798 33391921

[123] Goodyear E, Levin LA. Model systems for experimental studies: retinal ganglion cells in culture. Prog Brain Res. 2008;173:279–84. 18929116 10.1016/S0079-6123(08)01120-5

[124] Al-Ubaidi MR. RGC-5: are they really 661W? The Saga continues. Exp Eye Res. 2014;119:115. 24472671 10.1016/j.exer.2013.10.012 PMC3906592

[125] Sayyad Z, Sirohi K, Radha V, Swarup G. 661W is a retinal ganglion precursor-like cell line in which glaucoma-associated optineurin mutants induce cell death selectively. Sci Rep. 2017;7(1):16855. 29203899 10.1038/s41598-017-17241-0 PMC5715133

[126] Somvanshi RK, Zou S, Kadhim S, Padania S, Hsu E, Kumar U. Cannabinol modulates neuroprotection and intraocular pressure: A potential multi-target therapeutic intervention for glaucoma. Biochim Et Biophys Acta (BBA)-Molecular Basis Disease. 2022;1868(3):166325. 10.1016/j.bbadis.2021.166325 34921975

[127] Chen Q, Xi X, Zeng Y, He Z, Zhao J, Li Y. Acteoside inhibits autophagic apoptosis of retinal ganglion cells to rescue glaucoma-induced optic atrophy. J Cell Biochem. 2019;120(8):13133–40. 31021425 10.1002/jcb.28586 PMC6618276

[128] Hepler RS, Frank IR. Marihuana smoking and intraocular pressure. JAMA. 1971;217(10):1392. 5109652

[129] Imamura T, Tsuruma K, Inoue Y, Otsuka T, Ohno Y, Ogami S, Yamane S, Shimazawa M, Hara H. Rimonabant, a selective cannabinoid1 receptor antagonist, protects against light-induced retinal degeneration in vitro and in vivo. Eur J Pharmacol. 2017;803:78–83. 28315677 10.1016/j.ejphar.2017.03.018

[130] Imamura T, Tsuruma K, Inoue Y, Otsuka T, Ohno Y, Ogami S, Yamane S, Shimazawa M, Hara H. Involvement of cannabinoid receptor type 2 in light-induced degeneration of cells from mouse retinal cell line in vitro and mouse photoreceptors in vivo. Exp Eye Res. 2018;167:44–50. 29133122 10.1016/j.exer.2017.11.003

[131] Hepler RS, Petrus RJ. Experiences with administration of Marihuana to glaucoma patients. The therapeutic potential of Marihuana. Springer; 1976. pp. 63–75.

[132] Flach AJ. Delta-9-tetrahydrocannabinol (THC) in the treatment of end-stage open-angle glaucoma. Trans Am Ophthalmol Soc. 2002;100:215. 12545695 PMC1358964

[133] Tomida I, Azuara-Blanco A, House H, Flint M, Pertwee RG, Robson PJ. Effect of Sublingual application of cannabinoids on intraocular pressure: a pilot study. J Glaucoma. 2006;15(5):349–53. 16988594 10.1097/01.ijg.0000212260.04488.60

[134] Zhu Y, Zhou B, Chen X, Yao Y, Zeng Y, Zhang J, Cao Z, Ye Q, Zhang N, Yang J. MYOC/p. G367R mutation induces cell dysfunction of the trabecular meshwork and retina via impairment of the protein degradation mechanism. Res Square. 2022.

[135] Wang X, Yang Y, Li Y, Ba C. Acteoside attenuates retinal ganglion cell damage in acute high intraocular Pressure-Induced rats via the Bax/Bcl-xL axis. Med Res. 2022;6(1–2):220001.

[136] Hao H-J, Li Y-H, Yu B, Liu X, Zhang Y, Xing X-L. Neuroprotective effects of acteoside in a glaucoma mouse model by targeting Serta domain-containing protein 4. Int J Ophthalmol. 2024;17(4):625. 38638260 10.18240/ijo.2024.04.04 PMC10988069

[137] Fuller-Carter PI, Basiri H, Harvey AR, Carvalho LS. Focused update on AAV-based gene therapy clinical trials for inherited retinal degeneration. BioDrugs. 2020;34:763–81. 33136237 10.1007/s40259-020-00453-8

[138] Ryals RC, Boye SL, Dinculescu A, Hauswirth WW, Boye SE. Quantifying transduction efficiencies of unmodified and tyrosine capsid mutant AAV vectors in vitro using two ocular cell lines. Mol Vis. 2011;17:1090. 21552473 PMC3087449

[139] Kay CN, Ryals RC, Aslanidi GV, Min SH, Ruan Q, Sun J, Dyka FM, Kasuga D, Ayala AE, Van Vliet K. Targeting photoreceptors via intravitreal delivery using novel, capsid-mutated AAV vectors. PLoS ONE. 2013;8(4):e62097. 23637972 10.1371/journal.pone.0062097 PMC3637363

[140] Dalkara D, Kolstad KD, Caporale N, Visel M, Klimczak RR, Schaffer DV, Flannery JG. Inner limiting membrane barriers to AAV-mediated retinal transduction from the vitreous. Mol Ther. 2009;17(12):2096–102. 19672248 10.1038/mt.2009.181 PMC2814392

[141] Boye SE, Alexander JJ, Witherspoon CD, Boye SL, Peterson JJ, Clark ME, Sandefer KJ, Girkin CA, Hauswirth WW, Gamlin PD. Highly efficient delivery of adeno-associated viral vectors to the primate retina. Hum Gene Ther. 2016;27(8):580–97. 27439313 10.1089/hum.2016.085 PMC4991591

[142] Fuller-Carter PI, Basiri H, Harvey AR, Carvalho LS. Focused update on AAV-Based gene therapy clinical trials for inherited retinal degeneration. BioDrugs. 2020;34(6):763–81. 33136237 10.1007/s40259-020-00453-8

[143] Vandenberghe LH, Bell P, Maguire AM, Cearley CN, Xiao R, Calcedo R, Wang L, Castle MJ, Maguire AC, Grant R. Dosage thresholds for AAV2 and AAV8 photoreceptor gene therapy in monkey. Sci Transl Med. 2011;3(88):ra8854–8854. 10.1126/scitranslmed.3002103 PMC5027886 21697530

[144] Gonzalez-Cordero A, Goh D, Kruczek K, Naeem A, Fernando M, Kleine Holthaus S-M, Takaaki M, Blackford SJ, Kloc M, Agundez L. Assessment of AAV vector tropisms for mouse and human pluripotent stem cell–derived RPE and photoreceptor cells. Hum Gene Ther. 2018;29(10):1124–39. 29580100 10.1089/hum.2018.027

[145] Yang GS, Schmidt M, Yan Z, Lindbloom JD, Harding TC, Donahue BA, Engelhardt JF, Kotin R, Davidson BL. Virus-mediated transduction of murine retina with adeno-associated virus: effects of viral capsid and genome size. J Virol. 2002;76(15):7651–60. 12097579 10.1128/JVI.76.15.7651-7660.2002 PMC136354

[146] Allocca M, Mussolino C, Garcia-Hoyos M, Sanges D, Iodice C, Petrillo M, Vandenberghe LH, Wilson JM, Marigo V, Surace EM. Novel adeno-associated virus serotypes efficiently transduce murine photoreceptors. J Virol. 2007;81(20):11372–80. 17699581 10.1128/JVI.01327-07 PMC2045569

[147] Boye SL, Conlon T, Erger K, Ryals R, Neeley A, Cossette T, Pang J, Dyka FM, Hauswirth WW, Boye SE. Long-term preservation of cone photoreceptors and restoration of cone function by gene therapy in the guanylate cyclase-1 knockout (GC1KO) mouse. Investig Ophthalmol Vis Sci. 2011;52(10):7098–108. 21778276 10.1167/iovs.11-7867 PMC3207713

[148] Pang J-j, Dai X, Boye SE, Barone I, Boye SL, Mao S, Everhart D, Dinculescu A, Liu L, Umino Y. Long-term retinal function and structure rescue using capsid mutant AAV8 vector in the rd10 mouse, a model of recessive retinitis pigmentosa. Mol Ther. 2011;19(2):234–42. 21139570 10.1038/mt.2010.273 PMC3034861

[149] Gurtsieva D, Minskaia E, Zhuravleva S, Subcheva E, Sakhibgaraeva E, Brovin A, Tumaev A, Karabelsky A. Engineered AAV2. 7m8 serotype shows significantly higher transduction efficiency of ARPE-19 and HEK293 cell lines compared to AAV5, AAV8 and AAV9 serotypes. Pharmaceutics. 2024;16(1):138. 38276507 10.3390/pharmaceutics16010138 PMC10818700

[150] Wagner JE, Schön C, Becirovic E, Biel M, Michalakis S. In vitro evaluation of AAV vectors for retinal gene therapy. In: Weber BHF, Langmann T, editors. Retinal degeneration: methods protocols. New York: Humana Press; 2019. p. 383–90. 10.1007/978-1-4939-8669-9_24 30324456

[151] Böhm S, Splith V, Riedmayr LM, Rötzer RD, Gasparoni G, Nordström KJ, Wagner JE, Hinrichsmeyer KS, Walter J, Wahl-Schott C. A gene therapy for inherited blindness using dCas9-VPR–mediated transcriptional activation. Sci Adv. 2020;6(34):eaba5614. 32875106 10.1126/sciadv.aba5614 PMC7438099

[152] Bullock J, Pagan-Mercado G, Becerra SP. Cell-based assays to identify novel retinoprotective agents. MethodsX. 2020;7:101026. 32874942 10.1016/j.mex.2020.101026 PMC7452256

[153] Påhlman S, Ruusala A-I, Abrahamsson L, Mattsson ME, Esscher T. Retinoic acid-induced differentiation of cultured human neuroblastoma cells: a comparison with phorbolester-induced differentiation. Cell Differ. 1984;14(2):135–44. 6467378 10.1016/0045-6039(84)90038-1

[154] Gordon J, Amini S, White MK. General overview of neuronal cell culture. Methods Mol Biol. 2013;1078:1–8. 23975816 10.1007/978-1-62703-640-5_1 PMC4052554

